# Olivine-Carbonate Mineralogy of the Jezero Crater Region

**DOI:** 10.1029/2019je006011

**Published:** 2020-02-21

**Authors:** A. J. Brown, C. E. Viviano, T. A. Goudge

**Affiliations:** 1 Plancius Research, Severna Park, MD, USA,; 2 Johns Hopkins Applied Physics Laboratory, Laurel, MD, USA,; 3 Department of Geological Sciences, Jackson School of Geosciences, The University of Texas at Austin, Austin, TX, USA

## Abstract

A well-preserved, ancient delta deposit, in combination with ample exposures of carbonate outcrops, makes Jezero Crater in Nili Fossae a compelling astrobiological site. We use Compact Reconnaissance Imaging Spectrometer for Mars (CRISM) observations to characterize the surface mineralogy of the crater and surrounding watershed. Previous studies have documented the occurrence of olivine and carbonates in the Nili Fossae region. We focus on correlations between these two well-studied lithologies in the Jezero crater watershed. We map the position and shape of the olivine 1 μm absorption band and find that carbonates are found in association with olivine which displays a 1 μm band shifted to long wavelengths. We then use Thermal Emission Imaging Spectrometer (THEMIS) coverage of Nili Fossae and perform tests to investigate whether the long wavelength shifted (redshifted) olivine signature is correlated with high thermal inertia outcrops. We find that there is no consistent correlation between thermal inertia and the unique olivine signature. We discuss a range of formation scenarios for the olivine and carbonate associations, including the possibility that these lithologies are products of serpentinization reactions on early Mars. These lithologies provide an opportunity for deepening our understanding of early Mars and, given their antiquity, may provide a framework to study the timing of valley networks and the thermal history of the Martian crust and interior from the early Noachian to today.

## Plain Language Summary

We used Martian orbital data from a hyperspectral instrument on the spacecraft Mars Reconnaissance Orbiter (MRO) to assess the minerals on the surface of Mars at the future landing site of the Mars2020 rover. We found correlations between the minerals olivine and carbonate which are of interest to planetary scientists due to often being associated with volcanic activity (olivine) and life (carbonates). We used spectra of these minerals to infer the likely composition and grain size range of the olivine unit, and we document our new approach. We use our curve fitting approach to identify for the first time a special group of olivine-bearing rocks at Nili Fossae that show the most extremely redshifted absorption bands, and we show herein that these special olivines are not spatially associated with carbonates or clay rocks.

## 1. Introduction

### 1.1. Martian Carbonate Deposits

In the Martian context, it is the relative absence of carbonate over most of the globe that makes the mineral so intriguing. Carbonates were predicted to be present on Mars as large sedimentary deposits and have been sought by remote sensing studies as evidence of basaltic weathering under a greenhouse atmosphere in Mars’ distant past. Pollack et al. (1987) ran a 1D climate model of Mars under a CO_2_-rich atmosphere and found the only way for Mars to warm early in its history and retain liquid water on the surface was to somehow form a 1–5 bars CO_2_ atmosphere and thus create a global greenhouse. They used a terrestrial silicate weathering model to show that this atmosphere would likely disappear through weathering of basaltic material on the surface, creating abundant carbonate-bearing deposits on the surface. Pollack et al. also suggested that to extend the lifetime of the thick atmosphere, the carbonate rocks may have been recycled back into the atmosphere either through burial and thermal decomposition or direct decomposition through contact with hot lava. They stated, “A test of this theory will be provided by future spectroscopic searches for carbonates in Mars’ crust.” Recent studies (Bandfield et al., 2003; Edwards & Ehlmann, 2015) have placed some limits on the amount of carbonate exposed on the surface of Mars as detected by current orbital instruments, and small amounts of carbonates have been found in the Comanche rock at Gusev crater (Carter & Poulet, 2012; Morris et al., 2010) and recently studied in weathering profiles around Mars (Bultel et al., 2019); however, the hypothesized abundant carbonate-bearing deposits remain elusive.

The largest surface exposure of carbonate-bearing material identified to date was discovered in the Nili Fossae region using the CRISM instrument on the MRO spacecraft (Ehlmann et al., 2008). The origin of these carbonate deposits has been the subject of continuing debate in the Martian scientific community (Niles et al., 2012; Wray et al., 2016). The carbonate is always associated with an olivine spectral signature, as indicated by a wide 1 μm band, and we will therefore use the term “olivine-carbonate lithology” to describe this mineralogical association. The carbonate absorption bands also often occur with phyllosilicate Mg/Fe-OH hydroxyl bands (Brown et al., 2010; Goudge et al., 2015). Brown et al. (2010) showed that the carbonates in the Jezero crater region were most likely not pure Mg endmembers. They showed that their characteristic bands lie in the spectral region between Mg and Fe carbonates (see in particular their Figure 6) and away from the calcite spectral region, and for that reason, we refer to them as “Mg/Fe carbonates” in this paper.

The consistent co-occurrence of Nili Fossae carbonates and Mg/Fe-phyllosilicates could have many physical explanations. Ehlmann et al. (2009) suggested that low grade metamorphic or hydrothermal alteration in neutral to alkaline conditions may have formed these assemblages. Serpentinization is an especially promising method of simultaneous formation of carbonate and Mg/Fe-phyllosilicate (typically serpentine/greenalite or talc/minnesotaite) (Brown et al., 2010; Klein et al., 2013). Viviano et al. (2013) discovered that these assemblages (as defined in McSween et al., 2014) are limited to the eastern Nili Fossae region and suggested that they may have formed through continued alteration via high temperature (>200 °C) or low temperature (≤200 °C) fluid-limited carbonation of serpentine to form magnesium carbonate and talc-bearing material, in contact with the underlying Noachian mixed layer clay. McSween et al. (2014) pointed out that at the expected alteration temperatures of <350 °C, serpentine is favored for low CO_2_ activity, whereas talc is stable at higher CO_2_ activity (>0.1) in the alteration fluid. Higher CO_2_ activity tends to produce large amounts of carbonate in the final serpentinization assemblage (Greenwood, 1967; Hemley et al., 1977). Ehlmann et al. (2010) used CRISM data to find indications of serpentine across the planet. They located serpentine in Claritas Fossae, a number of southern highlands craters, and the Nili Fossae olivine-bearing lithology. Amador et al. (2018) recently conducted a search for serpentinizing minerals using a globally distributed subset of the CRISM dataset and showed that these assemblages are restricted to eastern Nili Fossae in association with the olivine-carbonate lithology.

Four related studies have invoked a shallow subsurface, low temperature emplacement of carbonate on Mars using CO_2_-rich brines. Niles et al. (2005) studied carbonate isotope ratios from ALH84001 and presented two models of CO_2_-bearing fluids traveling into the subsurface in order to create brine-like conditions that subsequently form carbonate rocks, invoking fluid mixing and an atmospheric CO_2_ origin to explain their isotopic observations. Michalski and Niles (2010) presented evidence of layered phyllosilicates and carbonates in Leighton Crater and suggested that they may have been buried by Syrtis Major lavas and then excavated from 6 km deep and may represent a regional subsurface carbonate reservoir. Glotch and Rogers (2013) conducted a study of the Thermal Emission Spectrometer (TES) dataset searching for breakdown products of carbonates, specifically creating an index for identification of the Ca-carbonate breakdown product portlandite. Their automated factor analysis based search achieved the best matches in the Nili Fossae region. Following the ideas of Michalski and Niles, they proposed a subsurface formation model that was driven by heat from Syrtis Major lavas, resulting in thermal metamorphism and assimilation of the carbonates and eventually production of portlandite. Finally, van Berk and Fu (2011) produced a dynamical geochemical model to study the subsurface layering caused by low temperature reactions under a thick CO_2_ atmosphere. This model was later used by Edwards and Ehlmann (2015) to suggest that low temperature carbonate sequestration was not likely to have removed sufficient CO_2_ from an early Mars atmosphere capable of causing a global greenhouse. This study is designed to bring to bear further observational data capable of critically testing these carbonate formation models.

### 1.2. Nili Fossae and the Jezero Crater Paleolake

**Figure 1.**
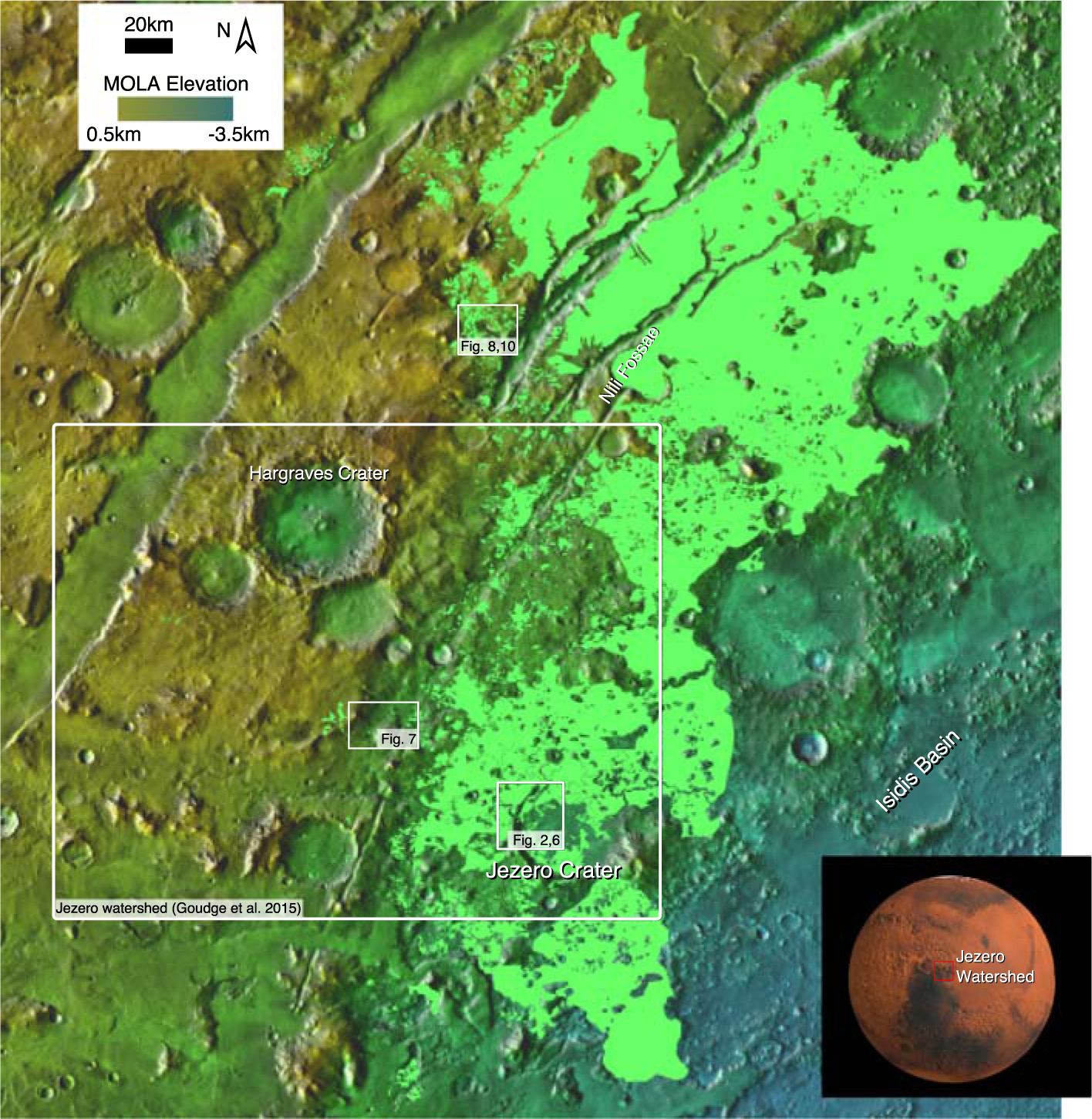
Location of Nili Fossae and Jezero crater (west of Isidis Basin) showing regions discussed in the text. Base map is “THEMIS Day IR with MOLA Color” obtained using JMars (Christensen et al., 2009). Inset image shows the study region in a red box, with the location shown as relatively dark and dust free. Shown in pale green is the extent of the olivine-carbonate unit as mapped by Kremer et al. (2019).

This study concentrates on the ancient Nili Fossae region of Mars, which is located adjacent to the Isidis impact basin (Figure 1). The peak of large basin formation in Martian history is estimated at between 4.1 and 4.25 Ga (Frey, 2008). The Isidis impact basin, at 1,350 km across, is the youngest large basin currently recognized and is dated at 3.96 Ga using observed crater retention age and Hartmann-Neukum model ages (Werner, 2008). The Noachian impact of the Isidis bolide created concentric features in the Martian upper crust that were discovered by Mariner 9 (McCauley et al., 1972) and soon thereafter were confirmed to be impact-related features (Wilhelms, 1973). The Isidis multi-ring impact structures are represented today as massive troughs that are exposed to the northwest of the Isidis Planitia basin (Schultz & Frey, 1990). These features have been modeled as relaxation graben structures caused by the weight of the Isidis mascon (Comer et al., 1985; Ritzer & Hauck, 2009). The features are known as the Nili Fossae, and they give their name to this well-exposed, largely dust-free chunk of Noachian crust in which they lie. The low relief, early Hesperian shield volcano of Syrtis Major lies to the south, and its lavas onlap the southern Noachian exposures of the Nili Fossae and the rim of the Isidis Basin (Hiesinger & Head, 2004).

Among the sites of interest in the Nili Fossae region is the paleolake basin contained within the ~45 km diameter Jezero impact crater (Fassett & Head, 2005). The Jezero crater paleolake is classified as hydrologically open and was fed by two inlet valleys to the north and west and drained by an outlet valley to the east (Fassett & Head, 2005). Buffered crater counts of inflowing valley networks indicate that this system ceased fluvial activity by approximately the Noachian-Hesperian boundary (Fassett & Head, 2008), similar to the timing of other large valley network systems on Mars (Fassett & Head, 2008; Hoke & Hynek, 2009). Jezero crater contains two well-exposed fluvio-lacustrine delta deposits (Ehlmann et al., 2008; Fassett & Head, 2005; Goudge et al., 2017, 2015; Schon et al., 2012) as well as large exposures of both phyllosilicate minerals and carbonates (Ehlmann et al., 2009; Ehlmann, Mustard, Fassett, et al., 2008; Ehlmann, Mustard, Murchie, et al., 2008; Goudge et al., 2017, 2015). In situ exploration of this region could provide novel insights into the fluvial sedimentary record, the timing of the valley networks, and the aqueous alteration history of early Mars. These outcrops contributed to the selection of Jezero crater in November 2018 as the landing site for the Mars2020 rover (Williford, 2018).

**Figure 2.**
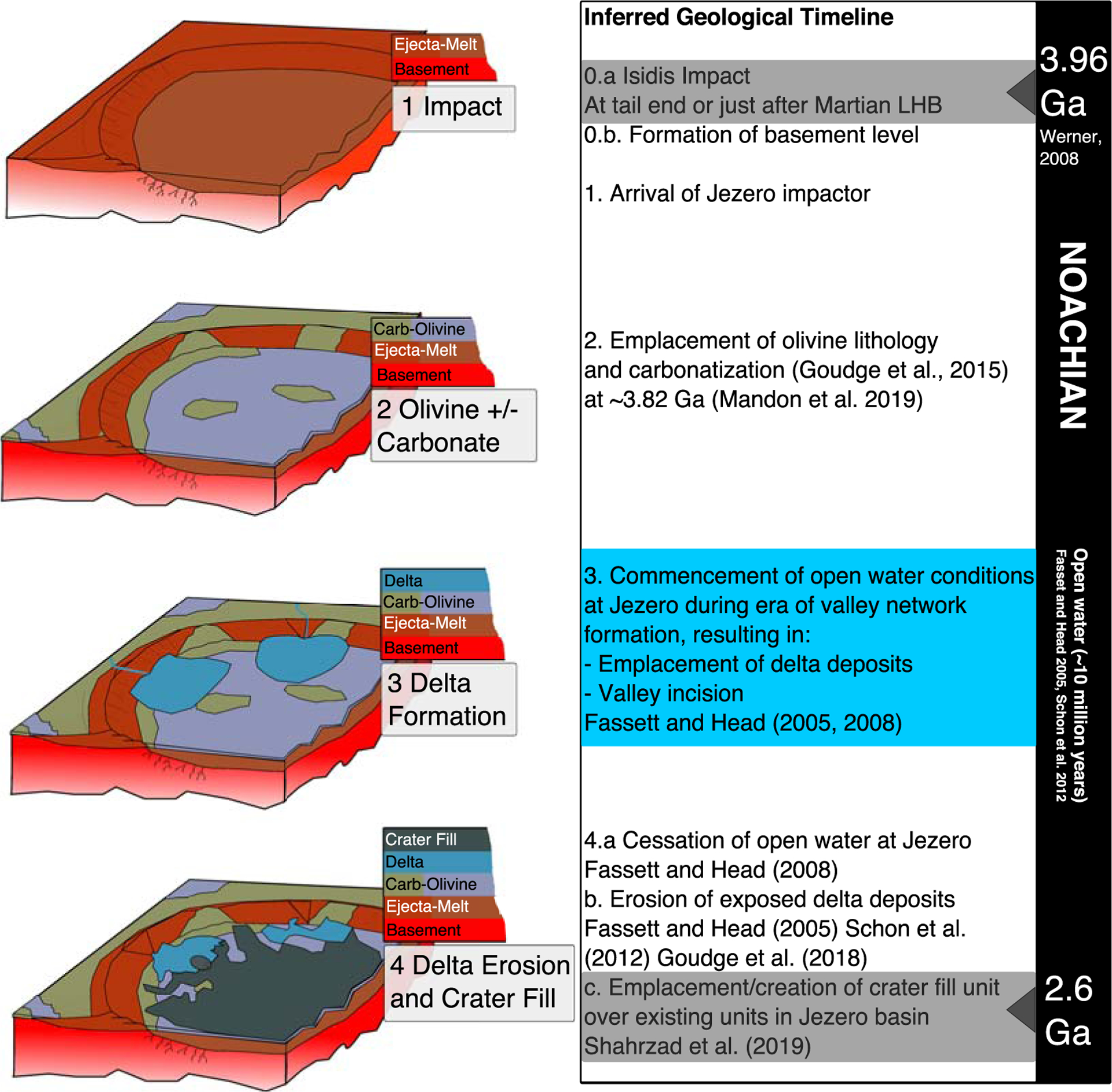
Framework formation history for Jezero crater lithologies for the period 3.96–2.6 Ga. Step 1 is the impact that formed Jezero, Step 2 is the emplacement of the Fe-rich olivine-carbonate lithology and the variable carbonatization of this lithology, Step 3 is the emplacement of the two deltas, and Step 4 is erosion of the deltas and emplacement of crater infill, leading to the crater as we see it today. Age of Isidis impactor is from Werner (2008), and age of crater fill unit is constrained by the crater counting study of Shahrzad et al. (2019).

An idealized geological sequence of events at Jezero crater is shown in Figure 2. This figure outlines our current interpretation of the timing of major geological processes that have shaped the crater and surrounding watershed as summarized in four steps. Step 1 is the impact that formed Jezero Crater, which postdates the Isidis basin formation and formation of megabreccia in the Nili Fossae regional basement unit (Mustard et al., 2009). Step 2 is emplacement of an olivine-bearing lithology and carbonatization of this lithology (Ehlmann, Mustard, Murchie, et al., 2008; Mustard et al., 2005, 2007). Step 3 is the formation of fluvial valleys, filling of the crater with water, and the emplacement of deltaic deposits, during the Late Noachian-Early Hesperian valley network forming period (Fassett & Head, 2008; Irwin et al., 2005). Step 4 is the erosion of the delta deposits to their current degraded state and infill of the crater by the floor resurfacing unit (Goudge et al., 2015; Schon et al., 2012). The latter two steps postdate the carbonate formation, since the northern and western delta have been interpreted to contain detrital carbonate transported to the basin from the watershed (Goudge et al., 2015). The timing of all these events is bracketed by the formation of the Isidis impact which is dated at ~3.8 Ga (Frey, 2008) and the recently derived date of the crater fill unit at Jezero at ~2.6 Ga (Shahrzad et al., 2019). The actual dating of Steps 1–4 is of immense interest to the Martian community, particularly since the Jezero twin deltaic deposits are potentially coeval with the formation of planet wide valley networks (Fassett & Head, 2005, 2008), which may indicate the last period when the surface of Mars was warm enough for liquid water to flow (Kite, 2019).

The two delta deposits within Jezero contain Mg/Fe-phyllosilicate and carbonate in varying proportions, with the northern fan dominated by carbonate and the western fan dominated by phyllosilicate (Goudge et al., 2015). The provenance of the phyllosilicate and carbonate within the Jezero crater deltas can be traced to mineralogically similar protolith units within the watershed, which provides strong evidence that the alteration minerals were primarily emplaced by fluvial transport (Goudge et al., 2015). These deposits therefore have bulk compositions that integrate heavily altered Martian crust of the Nili Fossae region (Ehlmann et al., 2009; Goudge et al., 2015; Mustard et al., 2009) and offer an opportunity to examine a diverse array of alteration minerals. Furthermore, Jezero crater contains large exposures of olivine- and carbonate-bearing units on the floor of the crater, underlying the deltaic deposits and draping the interior rim of the crater (Ehlmann et al., 2009; Ehlmann, Mustard, Murchie, et al., 2008; Goudge et al., 2015). These deposits were interpreted by Goudge et al. (2015) to represent exposures of the regional olivine-carbonate-bearing unit observed elsewhere in Nili Fossae (Ehlmann et al., 2009; Ehlmann, Mustard, Murchie, et al., 2008) and are similar to carbonate-bearing geomorphic units mapped recently in a study of the neighboring northeast Syrtis Major region (Bramble et al., 2017). A recent study by Salvatore et al. (2018) used regional thermal infrared observations to derive modal mineralogy of the N.E. Syrtis and Jezero crater regions and derived average carbonate abundance (4–17%, their Figure 11) for 12 TES spectra collected over Nili Fossae. Finally, a study by Mandon et al. (2020) has used crater counts to estimate the age of the Nili Fossae unit at 3.82 ± 0.07 Ga.

### 1.3. Previous Studies of Martian Olivine

#### 1.3.1. Olivine Properties and Composition

Olivine, [(Mg,Fe)_2_SiO_4_], is a geologically significant primitive planetary mineral which exhibits a continuous solid solution between its Fe-rich endmember Fayalite (Fa) and Mg-rich endmember Forsterite (Fo). Fayalite is 10 times less stable than Forsterite to low temperature weathering, which is thought to be relevant to modern Mars (Stopar et al., 2006). Fa is denser than Fo, which may lead to enrichments of fayalite in lag deposits under density (hydrodynamic) sorting due to aeolian processes (Fedo et al., 2015). Spectroscopically, Fa is discriminable from Fo because the substitution of Mg^2+^ for Fe^2+^ in the M1 and M2 sites of olivine crystal structure changes the site configuration and distorts the shape and position of the absorption band in energy space (Burns, 1970; Isaacson et al., 2011; King & Ridley, 1987; Sunshine & Pieters, 1998). The occupancies of the M1 and M2 sites both affect the 1 μm band that we use in this study; therefore, the band distortion is a not straightforward shift to long or short wavelengths. In addition, Mn^2+^ substitution can take place in these locations (Burns & Huggins, 1972); however, we ignore that process for this study as Mn because although it is concentrated in some rocks at Gale crater (Lanza et al., 2014), it is a minor component (~0.4 wt % MnO) compared to Mg and Fe in most Martian basalts (Taylor & McLennan, 2009). As will be discussed below, the strongest factor in shifting the 1 μm band to shorter wavelengths is high Mg^2+^ content; the strongest factor pushing it to longer wavelengths is higher Fe^2+^ content.

#### 1.3.2. Orbital Detections of Olivine

Olivine was first directly observed in the Nili Fossae region by Hoefen et al. (2003). They identified the olivine 400 cm^−1^ (25 μm) band in several TES (3 × 5 km per pixel) spectra and showed that the 25 μm band shifts to longer wavelengths for higher Fe content (lower Fo#). These were then best matched to the spectra of the Fo66, Fo41, and Fo60 samples of King and Ridley (1987), and they noted that no very low iron olivine was mapped in the region. The 25 μm band was shown not to significantly shift for an olivine sample (Fo89) which was sieved and separated into grain size distributions of <60, 60–104, 104–150, and 150–250 microns (Hoefen et al., 2003).

Higher resolution THEMIS images (100 m per pixel) were used to map olivine distribution across a large swath of Nili Fossae and constrain the composition to be Fo68–91 (Hamilton & Christensen, 2005), indicating a compositional range that was more magnesian than the TES estimate. They also identified layering and digitate forms associated with this olivine unit and used this observation to infer a magmatic origin.

Koeppen and Hamilton (2008) developed a TES index to find the signature of five Fo# ranges in a filtered subset of the TES dataset using one grain size range, (710–1,000 microns), which they stated was appropriate to dark regions such as Nili Fossae according to the model results of Ruff and Christensen (2002), which suggested that this dark material was consistent with a larger grain size distribution. Koeppen and Hamilton also performed a 500 km^2^ “double scan average” to reduce instrumental noise over Nili Fossae and obtained a qualitative match for their Fo58–74 index. These maps showed some Fo75–100 detections in and around Nili Fossae; however, the authors stated that most of the spectra from the region appear to best match Fo58–74. Koppen and Hamilton concluded that their finding of relatively high Fe-olivine called into question the Elkins-Tanton et al. (2005) magma ocean model, which envisions an early crust with Mg-olivine lying above Fe-olivine. They put forward an alternate model that includes an extra “leftover magma” that would supply an Fe-rich liquid and form a secondary crust above the uppermost Mg-olivine layer from the hypothesized Martian magma ocean.

Previous studies using visible and near-infrared (0.35–5.1 μm) data collected by the Observatoire pour la Minèralogie, l’Eau, les Glaces et l’Activitè (OMEGA) instrument on Mars Express have determined the presence of olivine in the Nili Fossae region. Mustard et al. (2005) used OMEGA data to map olivine and pyroxene in the Nili Fossae region, noting variations in the apparent position of the olivine 1 μm band position, possibly due to variations in grain size. In a follow-up study using higher resolution CRISM data, Mustard et al. (2009) showed a fayalite-like spectrum from CRISM image FRT00003E12 in their Figure 3, and noted that large particle size and textural effects could create the observed broad 1 μm band absorptions.

A number of global surveys using the OMEGA dataset that have mapped olivine composition have also been completed. Poulet et al. (2007) developed a set of spectral parameters for the OMEGA global dataset and mapped an Fo and Fa endmember in a global map for the first time. They again emphasized that large grain size may redshift the olivine 1 μm band to longer wavelengths. They reported that the Fa endmember, while easy to detect, was generally limited in occurrence and areal coverage. At the global scale, it was predominantly detected in the Nili Fossae region (Poulet et al., 2007).

In a follow up to the Poulet et al. study, Ody et al. (2013) conducted a further global study using OMEGA spectral indices and divided their olivine detections into Type 1 and 2. Their Figure 4 shows that Type 1 olivine, which they described as small grain size or Mg rich, was far more common globally and did occur, to a limited extent, in the Nili Fossae region. Type 2 olivine, which they described as Fe rich and/or large grain sized, was easier to detect but was far more limited in extent and mostly found in the Nili Fossae region.

Clenet et al. (2013) conducted a survey of OMEGA data using the Modified Gaussian Method (MGM) (Sunshine & Pieters, 1998) to constrain the mafic mineralogy. They reported that locally, within Nili Fossae, Fe-rich olivine was present; however, their study used lower spatial resolution OMEGA data, and they were not able to detect or correlate the presence of carbonate with olivine. Clenet et al. (2013) found that, locally, band centers are shifted to longer wavelength which could indicate Fo25–40 compositions or larger grain size.

**Figure 3.**
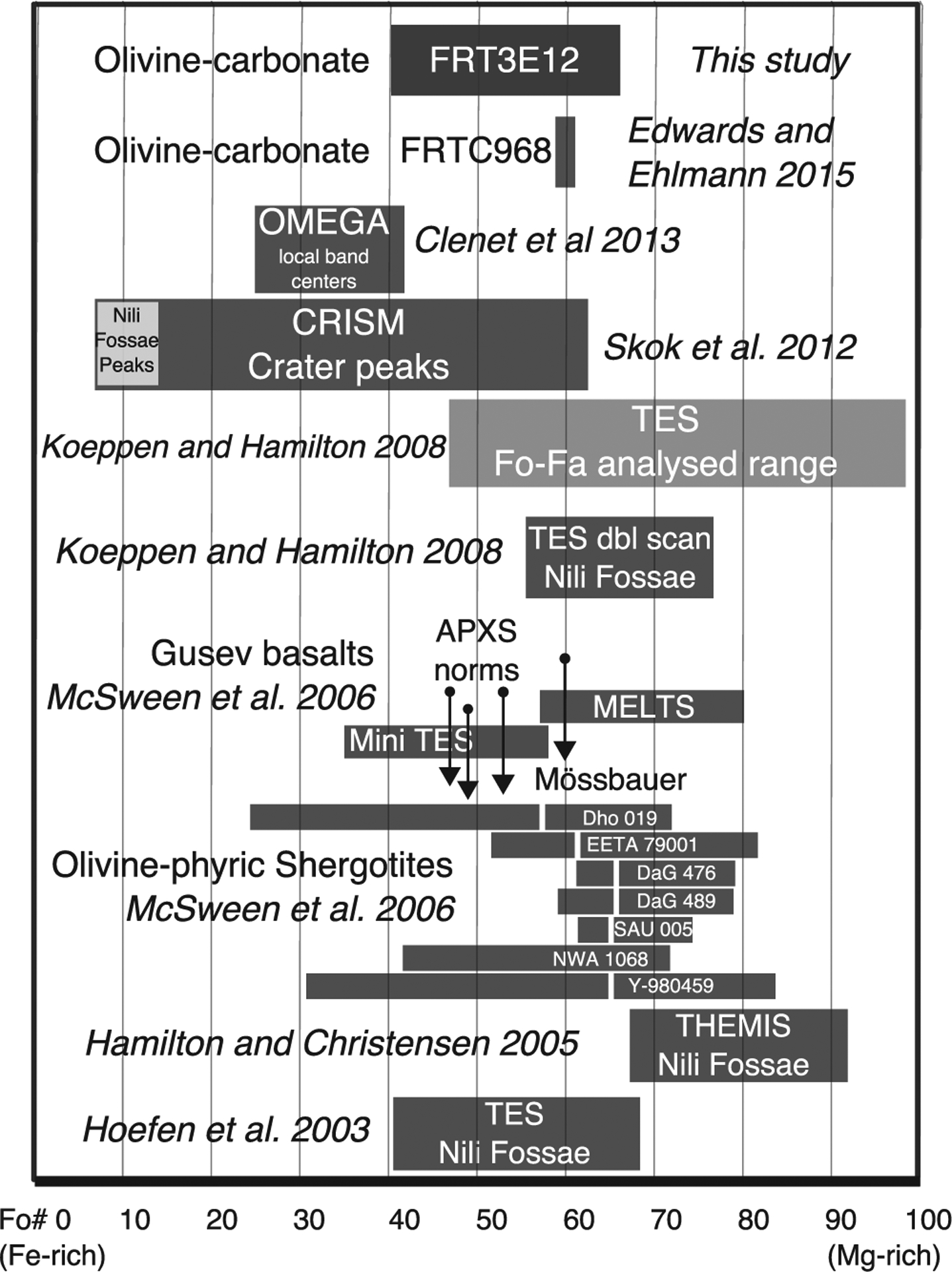
Summary of olivine composition results from previous remote and in situ studies of global Mars surveys, MER measurements at Gusev crater, and the olivine-phyric shergotites, updated, and expanded from McSween et al. (2006).

Skok et al. (2012) used the MGM approach to calculate the Fo# of CRISM observations of Martian impact crater central peaks, in order to access the most primitive Martian crust samples, and found a range of Fo5–60 in the craters they studied. At Hargraves crater and an unnamed crater, they found extremely iron-rich olivine compositions of Fo5–14. The olivine Fo# results we have discussed are summarized (where compositional estimates were provided) in Figure 3.

#### 1.3.3. In Situ and Meteorite Olivine Detections

Olivine-dominated (>20%) lavas (picrites) have been detected in situ by MER *Spirit* at Gusev crater (McSween et al., 2006). McSween et al. reported APXS estimates of the composition of the olivine as measured on basaltic “subcrop” rocks Adirondack, Humphrey, and Mazatzal as Fo52, Fo49, and Fo45, respectively. They used the pMELTS program to predict the original mantle olivine composition range of these three rocks and found it possible that these basalts were formed in a part of the mantle with Fo55–81 composition. The MER team also reported Mossbauer measurements that estimated the olivine composition as Fo60 (Morris et al., 2004). Finally, using the Mini-TES instrument, Christensen et al. (2004) provided an estimate of Fo35–60.

McSween et al. (2006) compared the findings of the MER team to the known Martian meteorites and determined that the most similar family are the olivine-phyric shergotitites. They pointed out that the shergotitites are too young to be linked to the ancient Columbia Hills basalts; however, they are the only meteorites family with (a) similar olivine phenocrysts, (b) similar olivine composition (Gusev: Fo40–60, olivine-phyric shergotites: Fo25–84 with norm of 65), (c) similar modal abundances (both 20–30 vol%), and (d) similar coexisting mineralogies. The olivine-phyric shergotitites compositional ranges are also summarized in Figure 3.

### 1.4. Outline of the Paper

The overarching goal of this study is to use CRISM data to map occurrences of previously detected carbonate and olivine deposits in order to determine whether any correlation exists at the 20 m scale between these two well-studied lithologies in the Nili Fossae region. We then assess whether any relationship exists between thermal inertia (using THEMIS IR data with pixel sizes of ~100 m on the ground) and the olivine 1 μm band position (detected by CRISM visible to near-infrared [VNIR] data with pixel sizes of ~18 m on the ground). We then summarize the potential effects of various physical factors on the 1 μm band position and draw conclusions from this. Finally, we study a variety of olivine and carbonate emplacement scenarios that have been proposed in the literature and determine which are more consonant with the mineralogical associations we have discovered.

## 2. Methods

We have analyzed data from the CRISM instrument (Murchie et al., 2007) covering the Jezero crater and the associated watershed for the presence of carbonate and olivine minerals exposed at the surface. A detailed list of CRISM observations and mineral identifications is listed in the supporting information accompanying this paper. CRISM has two primary operational modes, gimbaled hyperspectral (targeted: Full Resolution Targeted [FRT], Half Resolution Short [HRS], and Half Resolution Long [HRL]) and push broom multispectral mapping, that allow for targeted hyperspectral coverage over large areas of interest and global multispectral coverage at reduced spatial resolution. We use prototype or publicly available Map-projected Targeted Reduced Data Record (MTRDR) CRISM products for all full and half resolution images. These products have gone through a series of spectral corrections and spatial transformations to remove atmospheric absorptions and systematic noise and normalize the effects of atmospheric dust (Seelos et al., 2016).

Our approach to mapping olivine composition is in some ways related to previous studies of the olivine composition of the Moon using continuum removed M^3^ data (Isaacson et al., 2011) and follow-on laboratory studies of synthetic olivine samples (Isaacson et al., 2014). These studies have established the ability of continuum removed VNIR spectra to determine the relative Fe versus Mg composition from orbit and then checked their results using the MGM approach (Sunshine & Pieters, 1998). The MGM approach was specifically designed and tested on grain sizes <125 microns (Clenet et al., 2011; Sunshine & Pieters, 1993) and will not handle band saturation resulting from large grain sizes that are present in the CRISM dataset, which we document below. We are therefore required to develop alternative methods to constrain the olivine signatures in the olivine-carbonate lithology. Here the first attempt at such a model was described; however, it also has its shortcomings, and future models will no doubt improve on our results.

### 2.1. Olivine 1 μm Band and Asymmetric Band Fitting

In this study we have primarily used the asymmetric Gaussian spectral band fitting approach (Brown, 2006; Brown et al., 2010) to produce olivine-discriminating maps of the Jezero crater watershed. We summarize the key points of the model here, then discuss the parameters of this model and provide example asymmetric Gaussian fits. Further discussion of the parameters for each step is provided in the supporting information. The asymmetric Gaussian model achieves an iterative best fit using a Nelder and Mead (1965) simplex routine to optimally update the parameter set. The function to be fitted is an asymmetric Gaussian shape:

(1) $$\text{If}\;\lambda_{0}<\lambda f(\lambda )=\alpha \;exp\left(-{\left[\frac{\lambda -\lambda_{0}}{\sigma^{2}}\right]}^{2}\right)\;\text{if}\;\lambda_{0}\geq \lambda f(\lambda )=\alpha \;exp\left(-{\left[\frac{\lambda -\lambda_{0}}{\chi \sigma^{2}}\right]}^{2}\right)\;$$

**Figure 4.**
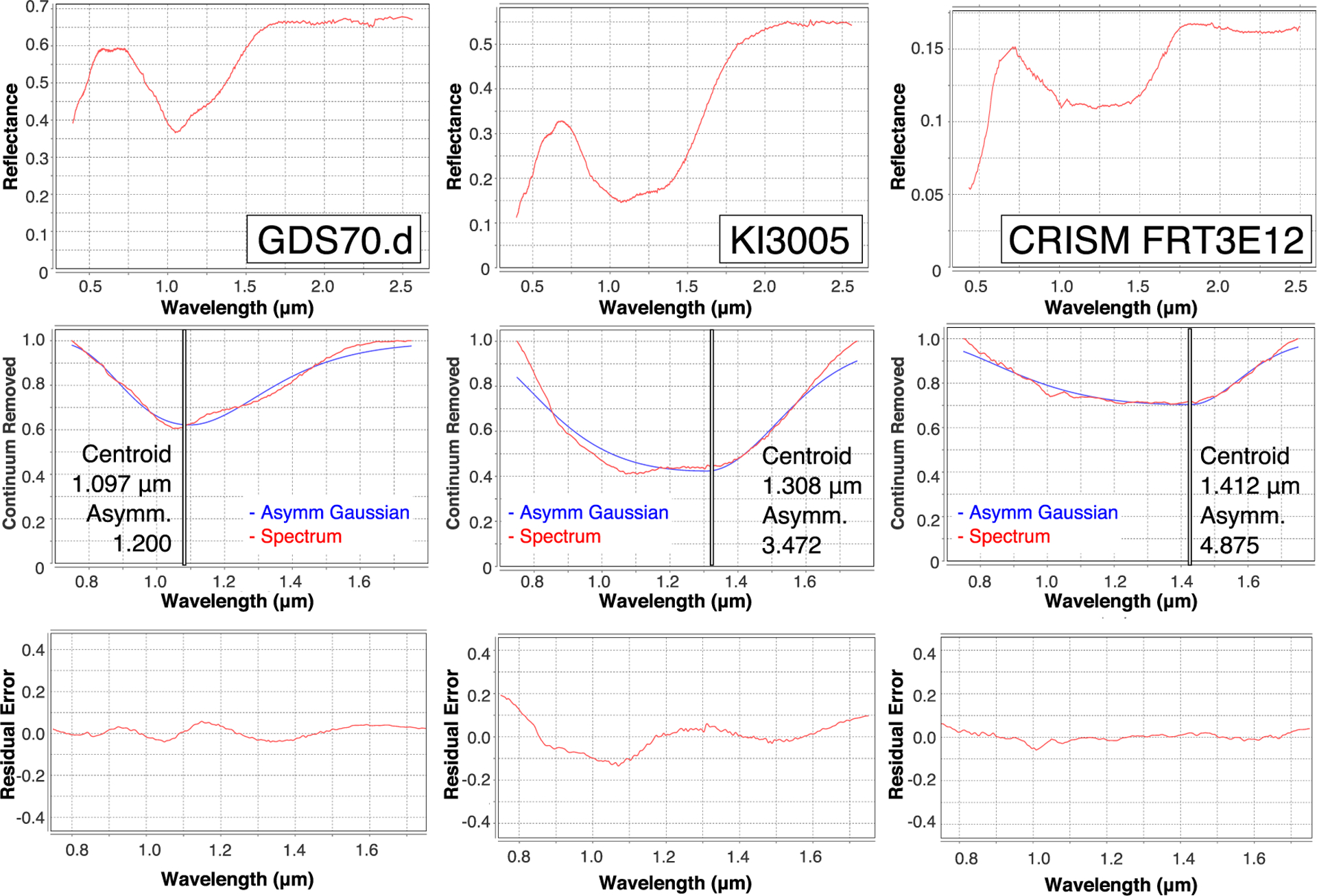
Example asymmetric Gaussian modeling of the library spectra of two laboratory olivine samples (GDS70.d and KI3005) and a redshifted olivine CRISM spectrum from FRT3E12. The GDS70.d has Fo#89, and the fitting band is more symmetric. The KI3005 has Fo#11, and the fitting band is more asymmetric. The CRISM spectrum 1 μm band is more saturated than the lab spectra, and so the centroid and asymmetry are even higher than the lab spectra.

The parameter set includes the centroid *λ*_*0*_, amplitude *α*, half width half maximum (HWHM) *σ*, and the asymmetry parameter *χ*. The asymmetry parameter is unbounded-values less than 1 indicate right asymmetry, and values greater than 1 indicate left asymmetry. All four of these parameters contain potentially useful information and can be used to discriminate bands of variable shape and strength from noise. We use the asymmetric Gaussian approach to model the 1 μm band of olivine, and we set the shoulders to be fixed at 0.7 and 1.75 μm throughout this study. We fit a straight line continuum between these two shoulder points, in order to reduce the number of fitting variables. A step by step plot of the process of fitting three olivine spectra (two laboratory spectra and a CRISM spectrum) with their asymmetric Gaussian fits is presented in Figure 4.

The position of the 1 μm olivine band is sensitive to the Mg versus Fe content, where more Fe-rich olivine displays a longer redshifted 1 μm band position (King & Ridley, 1987). In order to use the asymmetric Gaussian model to map the olivine 1 μm band (which is modelled in MGM by three overlapping Gaussian bands), we have to be judicious in our use of fit parameters and insert reality checks into the fitting process, while at the same time attempting to remove bias and consistently fit CRISM scenes that were taken under variable dust conditions and have some remaining amount of residual noise. We will discuss the external effects of variation of grain size and mixing with other components on this estimate in section 4 below.

We obtained seven Kiglapait (KI - Fo11–66) and four Green Sand Beach (GSB - Fo89) olivine spectra of varying composition and grain size from the study of King and Ridley (1987) from the USGS Spectral Library (Clark et al., 2007). In addition, we supplemented this collection of 11 USGS olivines with two additional USGS provided olivine spectra and eight additional olivines sourced from the RELAB reflectance database (http://www.planetary.brown.edu/relab/), in order to avoid biases due to reflectance measurements in a particular lab, as suggested by Trang et al. (2013).

**Figure 5.**
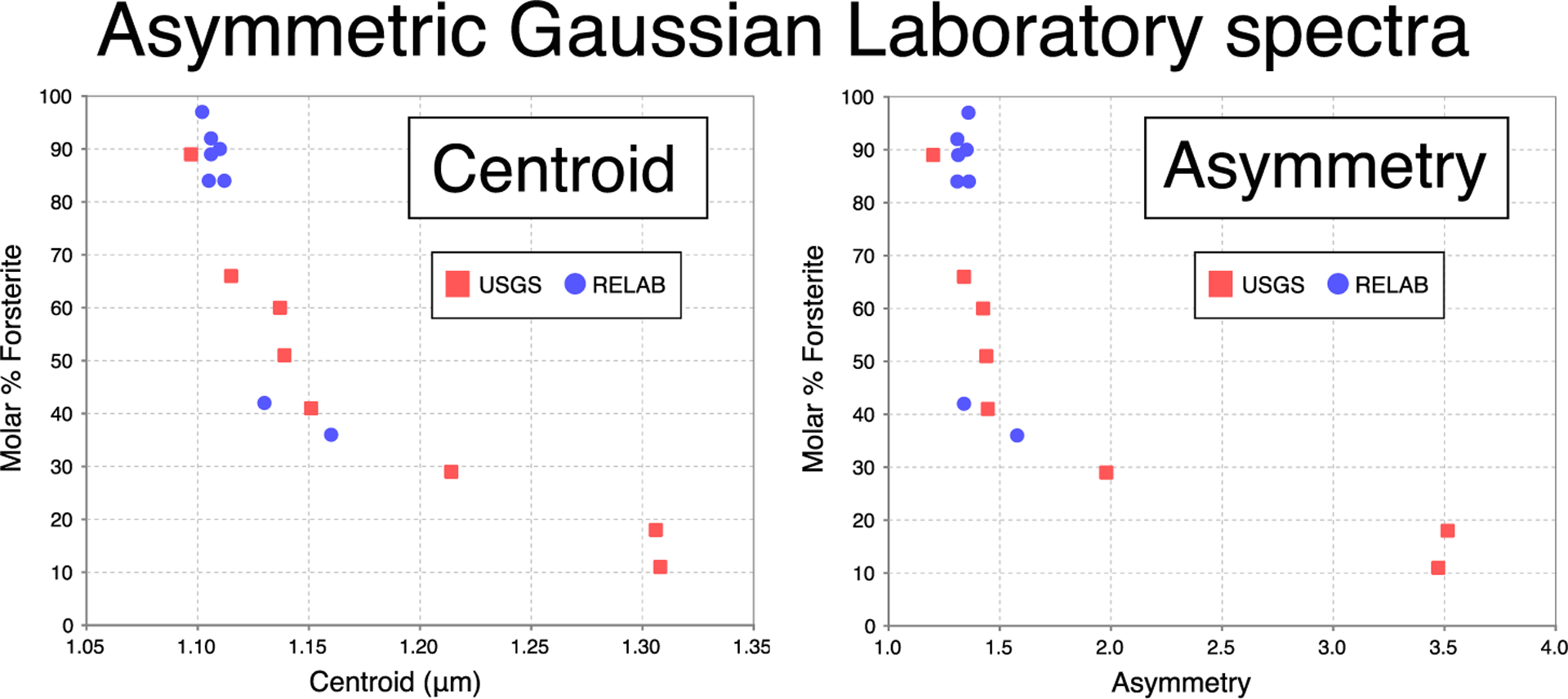
Laboratory spectra asymmetric Gaussian fitting results for RELAB and USGS spectra. These demonstrate the tendency of the centroid and asymmetry to decrease with increasing Fo#. This plot includes all 16 laboratory olivine spectra that have grain size <70 microns.

We have produced a plot of the 16 of the 21 olivine samples which have <70 micron grain size. Their measured centroid and asymmetry are shown in Figure 5. These plots show that for both RELAB and USGS laboratory olivine spectra, more Mg-rich samples have lower centroid and asymmetry, whereas more Fe-rich laboratory spectra have higher centroid and asymmetry values.

#### 2.1.1. Comparison of Laboratory Spectra to CRISM Spectra

The right column of Figure 4 shows an example CRISM olivine spectrum that has a 1 μm band that is saturated, which causes the band shape to flatten between 1 and 1.5 μm. For this spectrum, the asymmetric Gaussian method returns an asymmetry and centroid that are very high. In the middle laboratory spectrum (Fo#11, ~70 micron grain size), the 1 μm band is deeper than the CRISM spectrum; however, it is not as saturated, and this is reflected by it having lower centroid and asymmetry than the CRISM spectrum. The occurrence of the saturation and the manner in which it controls CRISM and laboratory spectra is the reason the asymmetric Gaussian method identifies olivines with high saturation, which are of key interest in this study.

#### 2.1.2. Rationale of Threshold and Subsetting Approach

The four parameters derived in the asymmetric Gaussian approach can all be combined to give a more robust estimate for the presence of olivine and its composition. For example, to reduce the chance of noisy spectra adversely affecting the results, a threshold can be placed on the height of the band and limits can be put on the asymmetry of the band. The choices we make reflect the noise structure and characteristics of the CRISM dataset and are the result of extensive iterative testing for the best parameters across the wide number of CRISM high resolution and mapping observations available to us at Nili Fossae. In order to restrict our mapping to the redshifted olivine lithology, and to reduce the probability of analyzing noisy spectra, we have thresholded the parameters of the resultant dataset as follows:

(2) $$\text{choose}\;\text{if}\;0.1<\alpha <1.0\;\text{and}\;1.15<\lambda_{0}<1.5\;\text{and}\;\sigma <0.3\;\text{and}\;0.8<\chi <9$$

where α is the amplitude, *λ*_*0*_ is the centroid, *σ* is the half width half maximum (HWHM), and *χ* is the asymmetry parameter. These parameters were chosen in a step-by-step variational approach using targeted and mapping CRISM data across Nili Fossae and were found to give the most uniform and robust results in mapping the olivine-carbonate lithology. This parameter filter has been applied to each CRISM dataset we discuss below in order to eliminate noisy and non-olivine-bearing pixels. By restricting the lowest admissible centroid to 1.15 μm, we have eliminated highly Mg-rich olivine (Figure 5) or fine-grained olivine spectra. This is done intentionally to focus the study on redshifted and saturated olivine signatures.

## 3. Results

Figure 1 shows the extent of the olivine-carbonate lithology as it has been mapped in previous studies (Bramble et al., 2017; Goudge et al., 2015; Mustard et al., 2005; Poulet et al., 2007). In order to establish the nature of the correlation between the olivine (mapped as either Fe- or large grain size olivine; Clenet et al., 2013; Ody et al., 2013) and associated carbonate units, we will now analyze three key regions with full resolution CRISM coverage from the Jezero crater, Jezero watershed, and just north of the Jezero watershed. The continuum removed example spectra, asymmetric Gaussian maps of olivine 1 μm position, carbonate maps, and correlation maps between 1 μm band position and asymmetry are provided for each key region.

### 3.1. Key Region 1: Olivine-Carbonate Correlations in Jezero Crater

**Figure 6.**
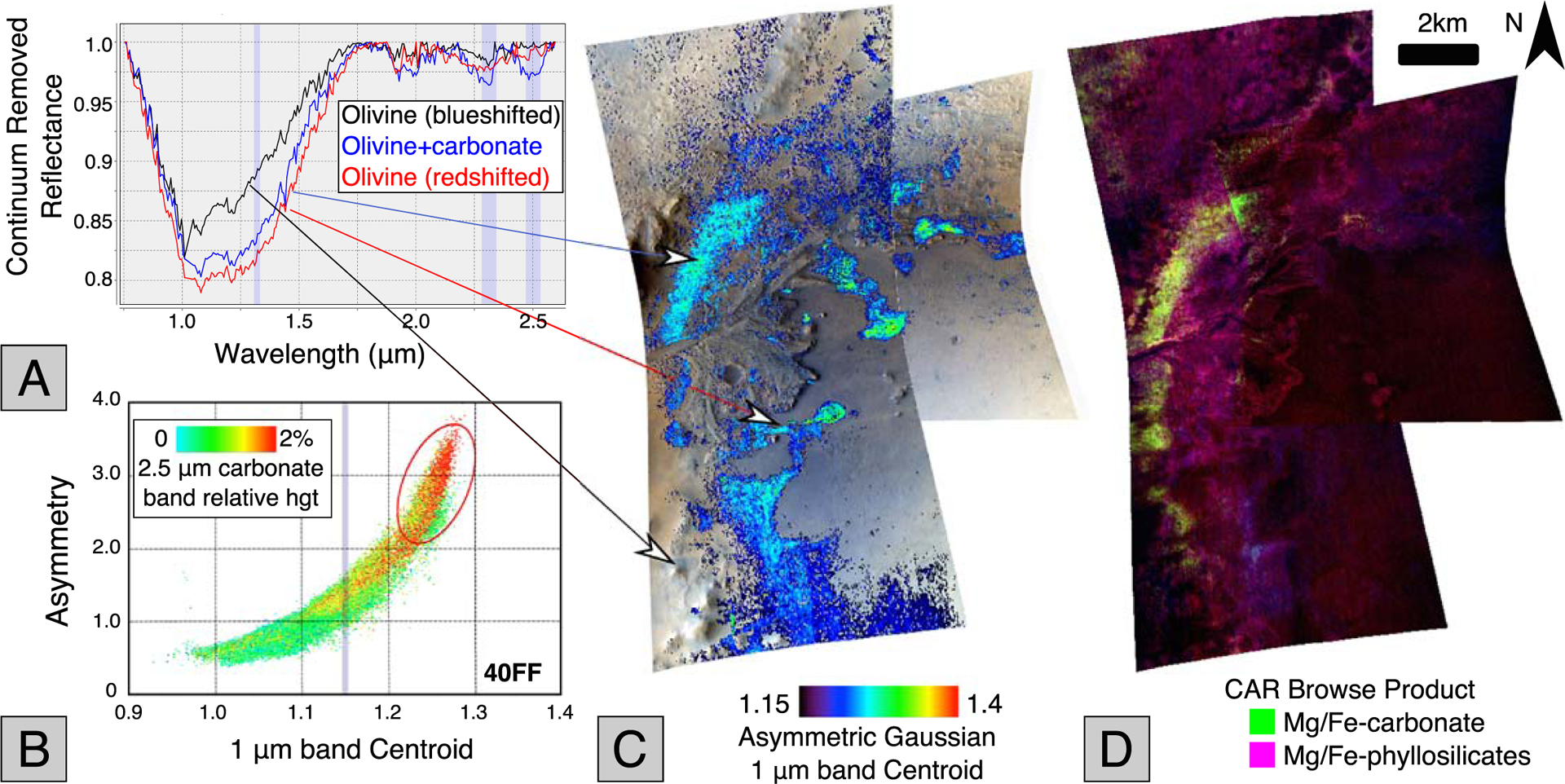
(a) Example continuum removed spectra from arrowed locations in images HRL40FF and FRT47A3. The 1.3, 2.3, and 2.5 μm regions are indicated by shaded vertical regions. (b) Plot of asymmetry versus centroid position for the 1 μm absorption band, color coded for the strengths of the 2.5 μm feature indicative of carbonates for all pixels from HRL40FF. (c) Olivine 1 μm band centroid map of Jezero western delta covered by CRISM images HRL40FF and FRT47A3. Arrows show regions where example spectra were obtained. (d) CRISM CAR standard browse product of the Jezero delta, (R: D2300, G: BD2500_2, B: BD1900_2) showing regions where carbonate is present due to the presence of a 2.3 accompanied by a 2.5 μm band (bright yellow-green-white tones). Phyllosilicates with 2.3 and 1.9 μm bands are in magenta.

Figure 6 presents our analysis results for two overlapping CRISM scenes over the western delta of Jezero crater. Figure 6a shows the type example continuum removed spectra at three representative points within the crater (locations shown by arrows in the figure). The continuum removed type spectra demonstrate the distinct shift in the wide olivine 1 μm band. The red spectrum represents the olivine unit that displays the most redshifted 1 μm band. The blue spectrum represents an example of an olivine-carbonate spectrum that is exposed on the west of the crater, as demonstrated by the accompanying carbonate bands at 2.3 and 2.5 μm (Gaffey, 1985, 1986, 1987; Hunt & Salisbury, 1971). The black spectrum corresponds to a lithology that contains the most blue shifted 1 μm olivine band, is sourced from the western rim of the crater, and displays no carbonate bands.

#### Carbonate correlation in Key Region 1.

Figure 6b shows a plot of the asymmetry versus the centroid for the olivine 1 μm band, both fit by the asymmetric Gaussian curve method for the HRL40FF CRISM image data shown in Figure 6c. The pixels in Figure 6b are color coded for the strength of the 2.5 μm band which is indicative of carbonates (red pixels show the strongest 2.5 μm band). This scatter plot clearly shows that there is spatial correlation between carbonates and redshifted 1 μm band centroid in the Jezero crater scene. The 1.15 μm cutoff for the threshold used in map Figure 6c is indicated by a vertical line. This shows that the large majority of the carbonates are located within the thresholded map; however, there are a small (<5%) number of carbonate (red) pixels below the threshold associated with olivine 1 μm bands that are below 1.15 μm.

Figure 6d shows the carbonate standard browse product (CAR) image, which highlights carbonate in green, Mg/Fe-phyllosilicates in magenta, and other hydrated minerals in blue (Viviano-Beck et al., 2014). This map demonstrates that the largest carbonate deposits in the crater are associated with the unit that is mapped in the thresholded 1 μm band centroid map in Figure 6c. It also shows variable carbonatization since there are olivine exposures to the east and south of the delta that are not associated with strong carbonate signatures.

### 3.2. Key Region 2: Partial Carbonatization of Olivine-Carbonate Lithology Across Nili Fossae

**Figure 7.**
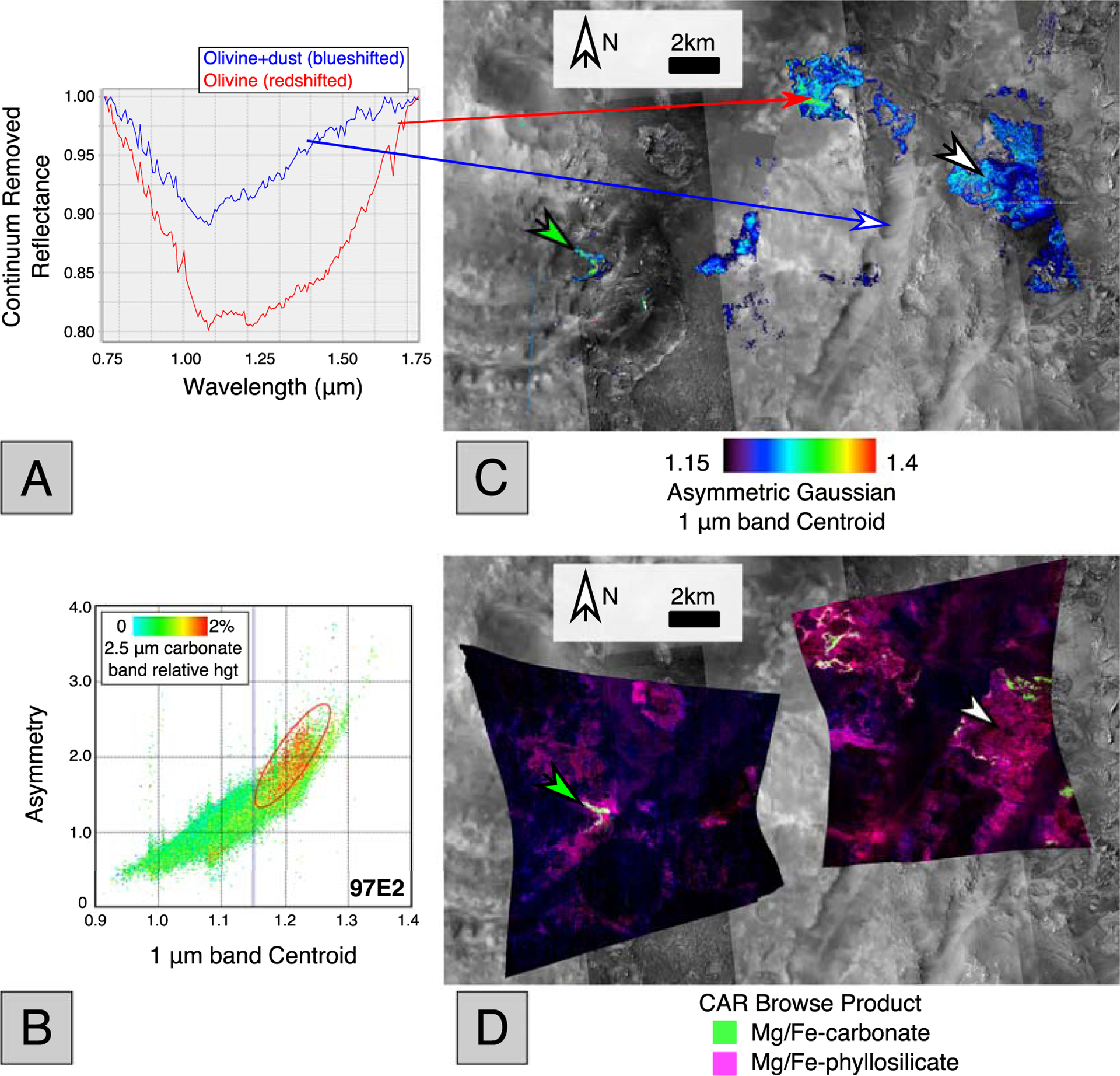
(a) Example continuum removed 1 μm band absorption band from two example points showing olivine (red) and dust with olivine (blue). The arrows indicate their locations on the CRISM image. (b) The correlation map of carbonate versus asymmetry and centroid, showing carbonate pixels (with a 2.5 μm band present) in red. The 1.15 μm threshold is indicated by a vertical line. (c) Olivine mineralogy of Nili Fossae as mapped in CRISM images FRT23370 (left) and 97E2 (right) overlying a CTX basemap with HiRISE images where available. The 1 μm band centroid map has been thresholded as discussed in the text. (d) Carbonate standard browse product (CAR) (R: D2300, G: BD2500_2, B: BD1900_2) showing regions where carbonate is present due to the presence of a 2.3 accompanied by a 2.5 μm band (bright yellow-green-white tones). Phyllosilicates with 2.3 and 1.9 μm bands are in magenta. Note that carbonates are only associated with the redshifted olivine unit, and this lithology is only partially carbonatized (green arrow indicates carbonate is detected, white arrows where it is not).

Figure 7 presents a CRISM image of a region over the Nili Fossae at the extreme western edge of the Jezero watershed (location in Figure 1). As for Figure 6, the CRISM 1 μm centroid map has been thresholded to a lower limit to isolate our analysis to only redshifted olivine-rich exposures.

#### Carbonate correlation in Key Region 2.

This figure demonstrates a key property of the carbonatization process with respect to the redshifted olivine-bearing lithology: The carbonate-bearing regions of this lithology are spatially variable. The green arrows on Figures 7c and 7d indicate that when the carbonate is present, it is located within a redshifted olivine lithology. The white arrows on Figures 7c and 7d show regions of the redshifted olivine-bearing lithology that have not been carbonatized (since this region is not green on Figure 7d).

The correlation plot in Figure 7b shows the location of carbonates in the asymmetry versus centroid space of the olivine 1 μm band for FRT97E2. This provides further evidence that for this image, the carbonates are grouped in a relatively constrained region of the plot, showing above average centroid and high asymmetry values. Two further points are worth noting here:

1. In this scene, not all redshifted olivine pixels are carbonate bearing. This spatial relationship is also manifested within Jezero Crater, where the redshifted olivine lithology is also variably carbonatized.
2. In this scene, in contrast to the last, the carbonates do not correspond to the absolute maximal extrema of centroid values in the image, which indicates variation in the olivine-carbonate spatial relationship between watershed and the delta region.

### 3.3. Key Region 3: Evidence for Olivine Band Saturation

**Figure 8.**
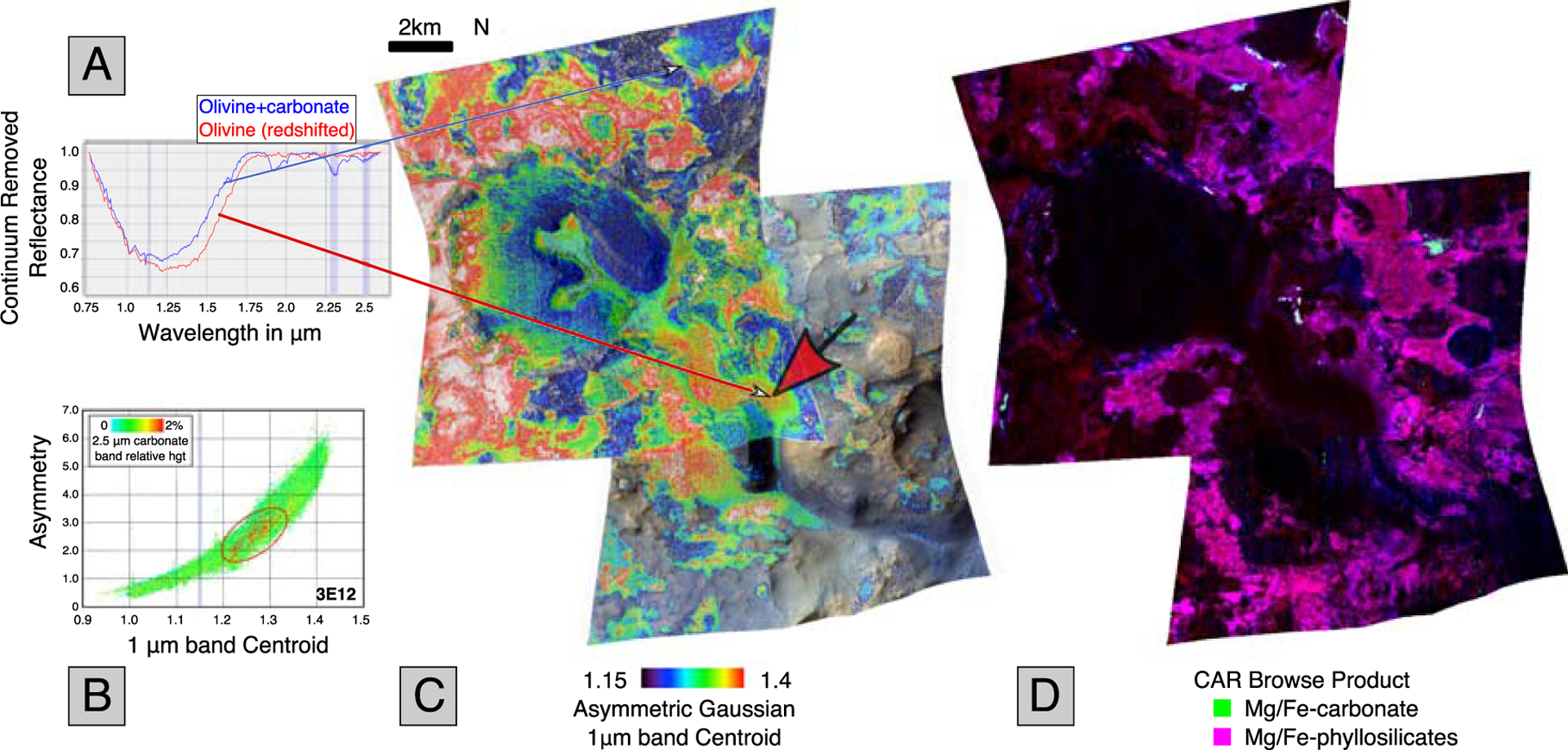
Olivine and carbonate mineralogy for CRISM image FRT3E12 and FRTB438. (a) Example continuum removed spectra from CRISM FRT3E12, with locations shown by arrows. Note that 2.3 and 2.5 bands indicate the presence of carbonate in the blue spectrum. The red spectrum does not have carbonates, and the 1 μm band is further redshifted to long wavelengths. (b) Asymmetry versus centroid correlation map for 3E12, color coded in red for the presence of the 2.5 μm carbonate band. The plot shows carbonates are not associated with the extreme right shifted olivine spectra in this scene. The 1.15 μm threshold is shown as a vertical line. (c) 1 μm band centroid map for FRT3E12 and FRTB438. The large red arrow indicates an area where dunes have formed that is also indicated by a red arrow in Figure 9. (d) CRISM CAR Browse product (R: D2300, G: BD2500_2, B: BD1900_2) showing regions where carbonate is present due to the presence of a 2.3 accompanied by a 2.5 μm band (bright yellow-green-white tones). Phyllosilicates with 2.3 and 1.9 μm bands are in magenta.

Figure 8 presents data for CRISM full resolution images 3E12 and B438, which are located at a re-entrant close to the Nili Fossae trough (see Figure 1 for the location). Figure 8a shows representative continuum removed spectra from the scene, including an example with 2.3 and 2.5 μm bands and one without. Both show redshifts of the 1 μm band; however, the one without carbonate bands is redshifted by a greater amount. Figure 8a also shows an example continuum removed spectrum in red that shows a flattening of the base of the band that we interpret as band saturation, caused by large grain size, and the same spectrum was analyzed in Figure 4.

#### Carbonate correlation in Key Region 3.

Figure 8b is a plot of asymmetry versus centroid position for the 1 μm band, with colors and an ellipse indicating where carbonate-bearing material plots. This demonstrates that in this scene, in contrast to the delta scene (Key Region 1), but in family with the watershed scene (Key Region 2), the carbonate is not associated with extremal shifts in the 1 μm band. Figure 8c shows the 1 μm band centroid images which demonstrate that olivine is present in great abundance in these images. Figure 8d shows that the image contains carbonates, mostly outcropping around the central horseshoe shaped feature (which is dark in Figure 8d) and exposed on the exterior of this extended mesa.

### 3.4. Correlations of 2.3 and 2.5 μm Band With 1 μm Band

We have seen that the carbonate 2.5 μm band is correlated in a slightly different way in each of the Key Regions 1 to 3. We wish to investigate the behavior of the 2.3 μm band in order to dig deeper into the relationship between olivine and other accessory minerals. The 2.3 μm band is present in carbonates but is also present in phyllosilicates (Hunt & Salisbury, 1971). Because both carbonates and phyllosilicates are present in the pixels of our scene, the 2.3 μm band signatures cannot be relied upon as a unique identifier of either mineral. However, the absence of a 2.3 μm signature is indicative of a lack of both carbonates and phyllosilicates. We will look for this in our correlation plots below.

**Figure 9.**
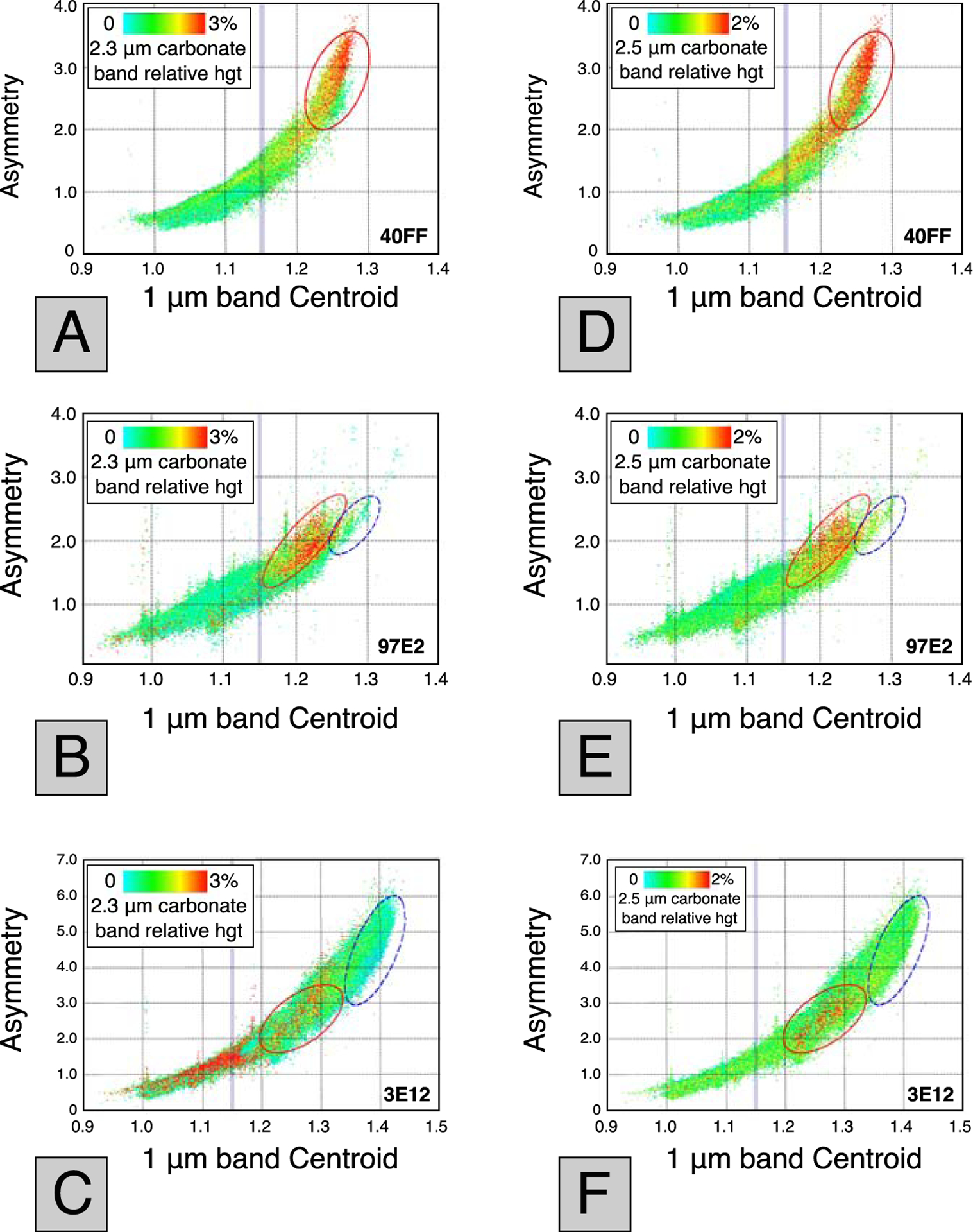
Asymmetry versus centroid correlation maps. The 1.15 μm threshold is shown as a vertical line. (a-c) Color coded in red for the presence of the 2.3 μm phyllosilicate + carbonate band, relative height scaled to 3%. (d-f) Color coded for presence of 2.5 μm carbonate band, relative height scaled to 2%. Red ellipses correspond to pixels that show both a 2.3 and 2.5 μm band. Blue (dashed) ellipses show absence of these bands at the longest band centers.

Figure 9 shows the asymmetry versus centroid plots for HRL40FF, FRT97E2, and FRT3E12, color coded for the 2.3 μm (Figures 9a–9c, left) and 2.5 μm (Figures 9d–9f, right) bands-the 2.5 μm plots are identical to Figures 6b,7b, and 8b. These plots were constructed by using a symmetric Gaussian band fitting procedure to identify the strongest band in the 2.1–2.4 μm and 2.4–2.6 μm region (Brown, 2006) and color coding each pixel by its relative height. The 2.3 μm plots are relatively scaled to 3% compared to 2% for the 2.5 μm band. Comparing the plots in the left column to those on the right, one can observe that the presence and distribution of a 2.5 μm band is always accompanied by a 2.3 μm band, as might be expected for carbonate minerals. Figures 9a and 9d display a relatively similar distribution. Figures 9b and 9e are also relatively similar; however, there are red pixels in the left half of the 2.3 μm plot that do not have a counterpart in the 2.5 μm plot, corresponding to the absence of carbonates and the presence of phyllosilicates. Figures 9c and 9f continue this trend to an even greater extent, and there are many red pixels in the 2.3 μm plot on the left or blue shifted part of the plot that have no corresponding 2.5 μm band in Figure 9f. This again indicates the presence of phyllosilicates and absence of carbonates.

The most important take away from Figure 9, as far as this study is concerned, is the relationship between the 2.3 and 2.5 μm bands with band centroids greater than 1.15 μm. In Figures 9a and 9c (HRL40FF), we first note the presence of 2.3 and 2.5 μm bands for the most redshifted pixels and contrast this with the relative absence of these bands from the right most (most redshifted) part of Figures 9d and 9f (FRT3E12). We have placed a blue (dashed) ellipse around these regions in 3E12 to highlight their importance. The absence of both 2.3 and 2.5 μm bands in these most redshifted regions is demonstrated by the green to blue color of these pixels. Overall, FRT97E2 and HRL40FF appear relatively similar; however, there are a small number of pixels in 97E2 where the same redshift and absence of 2.3 and 2.5 μm bands as in 3E12 happens, although to a lesser (and more tenuous) extent in Figures 9b and 9e, and we have placed a small blue ellipse around those regions of interest.

### 3.5. Summary of Olivine-Carbonate Correlations

In the Key Regions 1 to 3 just discussed, we have shown that the nature of the correlation between the olivine and carbonates is variable across the extent of the lithology. In the delta region of Jezero crater, Figure 6b shows that the 2.5 μm carbonate band is associated with the most extreme shifted olivine in that region. In Key Region 2, in the Jezero watershed, Figure 7b shows that there is a small amount of extreme redshifted olivine-bearing material that is not associated with carbonate bands (in the 1.25–1.28 μm centroid range). In Key Region 3, Figure 8b shows this behavior even more clearly. In that figure, the carbonates are in the 1.2–1.3 μm centroid range; however, there is a large amount of olivine material that is further redshifted into the 1.4–1.5 μm centroid range that is not associated with carbonates.

In all three regions, carbonate signatures are not associated with the blue end of the range (1–1.2 μm centroid range). There are no carbonate occurrences that are not associated with olivine 1 μm band complexes. This shows that the olivine units previously mapped as Fe- or large grain size (Clenet et al., 2013; Ody et al., 2013) are indeed associated with the carbonates mapped previously by Ehlmann, Mustard, Murchie, et al. (2008); however, the most extreme redshifted olivines are in fact not associated with carbonate spectral signatures. In addition, Figure 9 shows us that phyllosilicates are also absent from these most redshifted olivines, as shown by the lack of 2.3 μm bands in Figure 9c (highlighted by blue ellipse).

### 3.6. THEMIS Thermal Inertia Mapping

In order to develop a greater understanding of the lithologies we have chosen to focus on in this study, we will now examine the thermal inertia maps generated using THEMIS covering the same regions as our best exposed CRISM scenes shown in Figure 8. To first order, for unconsolidated material, higher thermal inertia corresponds to larger grain size (Fergason et al., 2006); and therefore, we wish to establish whether there might be a relationship between the olivine 1 μm centroid position and the thermal inertia of the corresponding location.

**Figure 10.**
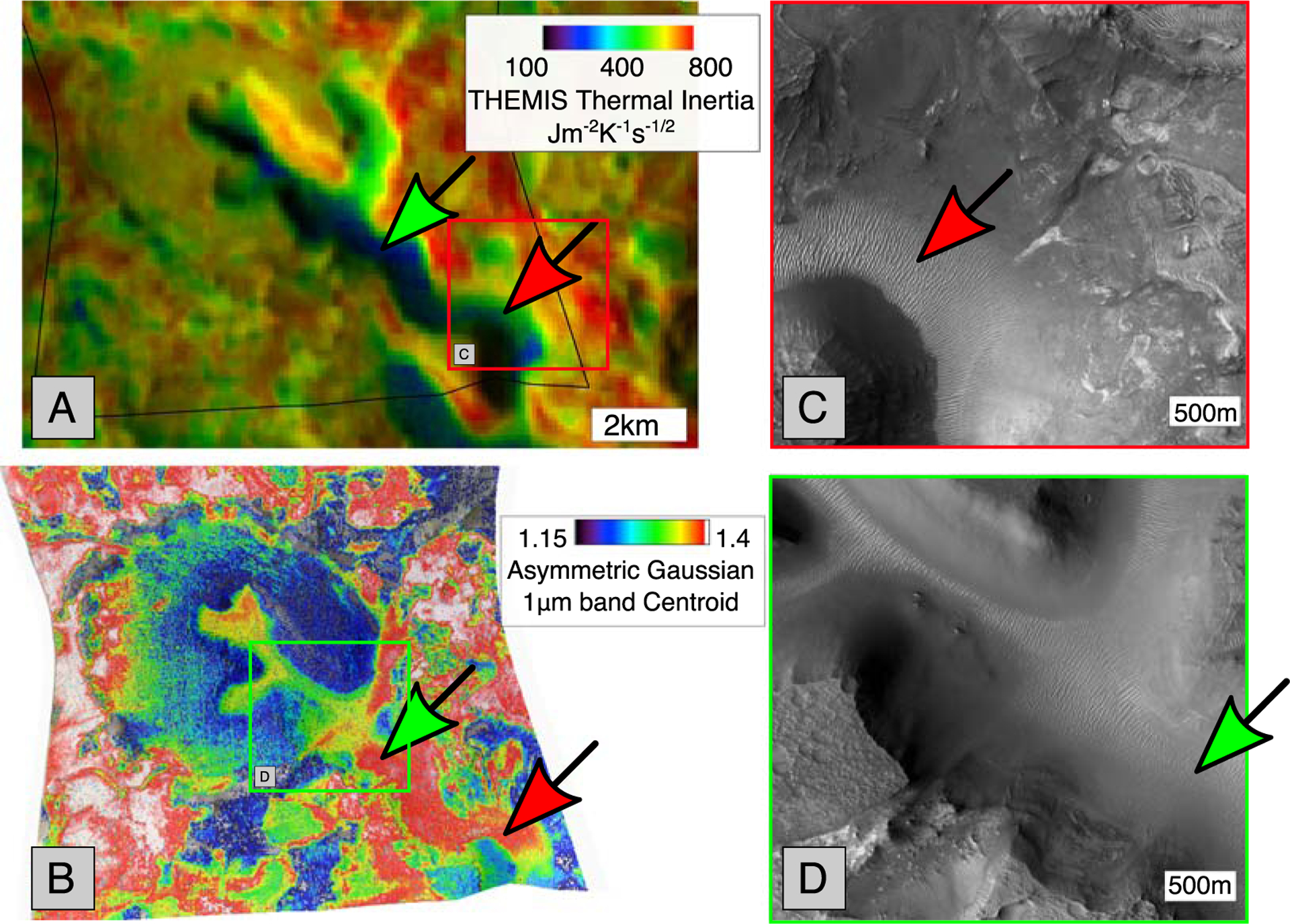
(a) THEMIS thermal inertia map around CRISM image FRT3E12 (outline shown in black). (b) CRISM image 3E12 1 μm band centroid image that overlaps the THEMIS scene, showing red and green arrows pointing to redshifted olivine. (c) HiRISE image ESP_026992_2025_RED magnified showing location of dunes pointed to by red arrow. (d) HiRISE image PSP_002888_2025_RED showing magnified location of green arrow pointing to dunes in the THEMIS image (a), blue represents low thermal inertia, fine grain material, red represents bedrock, large grain size material. The red and green arrows in (a) point to blue low thermal inertia material, implying a fine grain size. In the CRISM 1 μm band image, red (and white) indicates the highest redshifted olivine. In (b) the red and green arrows point to highly redshifted material.

Figure 10a presents the THEMIS thermal inertia map for the FRT3E12 region. In this image, blue corresponds to low thermal inertia (more fine grained material) and red corresponds to high thermal inertia (typically larger grained or bedrock). The large red arrow (also shown in Figure 8) indicates an area where dunes can be seen to have formed (shown in HiRISE in Figure 10c), and this area displays correspondingly low thermal inertia and hence low grain size. Figure 8a shows a CRISM spectrum (in red) taken from the area marked by the red arrow. This spectrum shows that this low thermal inertia region is covered by redshifted olivine material.

Figure 10b shows the CRISM 1 μm centroid map, with the red and green arrows pointing to red regions, which again indicates that this is some of the most redshifted olivine material in the scene. In Figure 10d, the location of the green arrow is shown in HiRISE, again showing the presence of dunes in the location of the most redshifted material.

Some regions in Figure 10a (e.g., on the western edge) show high thermal inertia (Figure 10a in red) and high redshifted material (Figure 10b in white), and these locations display what we might term a normal correlation between thermal inertia and redshift. However, the dune-covered channel in the southeast of the image (indicated by the green and red arrows) shows an abnormal correlation, with low thermal inertia but a high redshift. We will address the possible interpretations of this observation in the discussion (section 4.3) below.

## 4. Effects of Mineralogy, Dust, and Grain Size

### 4.1. Potential Effects on Olivine 1 μm Band Position

The main findings of this study rest upon apparent shifts of the central position and asymmetry of the 1 μm band complex due to olivine. This band has been documented to apparently shift to low or high wavelengths for a variety of physical scenarios (Cloutis et al., 1986; Dyar et al., 2009; Mustard et al., 2005; Poulet et al., 2007), some of which are relevant to this study of Martian olivine composition. It should be noted that the olivine 1 μm band complex does not exhibit a shift in band centroid as temperature decreases to 80 K (Singer & Roush, 1985); therefore, we do not consider the effect of temperature changes in this study. We first examine the effect of mixing with pyroxene, then dust, then carbonates, then synthetic olivine, and finally consider the effect of changes in grain size of the olivine sample.

#### 4.1.1. Effects of Intimate Mixing With Pyroxene

As with any remote sensing study, we must consider the effect of contaminants and how they may affect our band fitting procedures. In order to determine the potential effects of mixing on centroid and asymmetry ranges of the 1 μm band, we applied the asymmetric Gaussian fitting algorithm described earlier to a range of spectra that include progressively larger amounts of pyroxene mixed with olivine.

**Figure 11.**
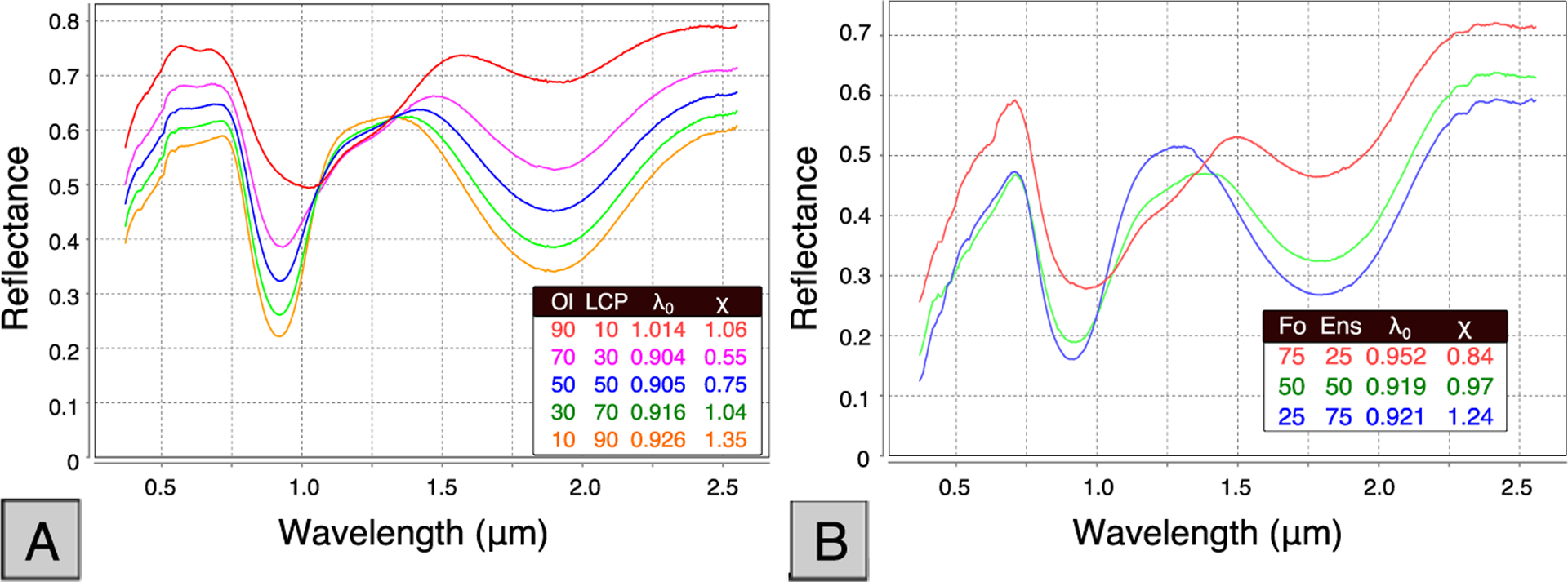
Effects on 1 μm band position of intimate mixing of olivine with pyroxene, showing a shift to shorter wavelengths with greater amounts of pyroxene. (a) Intimate mixing of olivine with low calcium pyroxene (LCP) from Corrigan et al. (2007). (b) Intimate mixing of Forsterite olivine and Enstatite pyroxene, data from Freeman et al. (2010).

In Figure 11 we show plots of two studies of mixing of olivine and pyroxene that we obtained from RELAB. These studies were carried out by Corrigan et al. (2007) (Figure 11a) and Freeman et al. (2010) (Figure 11b). Figure 11a shows that the presence of pyroxene has the effect of moving the 1 μm band to the left (shorter wavelengths). Corrigan et al. used the San Carlos olivine which is ~Fo84.

In order to determine the magnitude of this effect and how it would affect our study, we first fitted the 1 μm band of the red spectrum in Figure 11a, which has the smallest amount of pyroxene (90/10). Our fit for the 90/10 mixture revealed that the centroid of the 1 μm band was at 1.014 μm. In addition, the asymmetry of the 1 μm band for the 90/10 spectrum was found to be 1.06. We then carried out a fit of the orange spectrum, which is 90% pyroxene and 10% olivine. The fit for the 10/90 mixture gave a centroid of 0.926, which results in a maximal potential shift of 88 nm for pyroxene mixing in the 90% to 10% range available with this dataset. The asymmetry for the 10/90 mixture was 1.35, for a difference of 0.29.

#### 4.1.2. Effects of Linear Mixing With Dust

**Figure 12.**
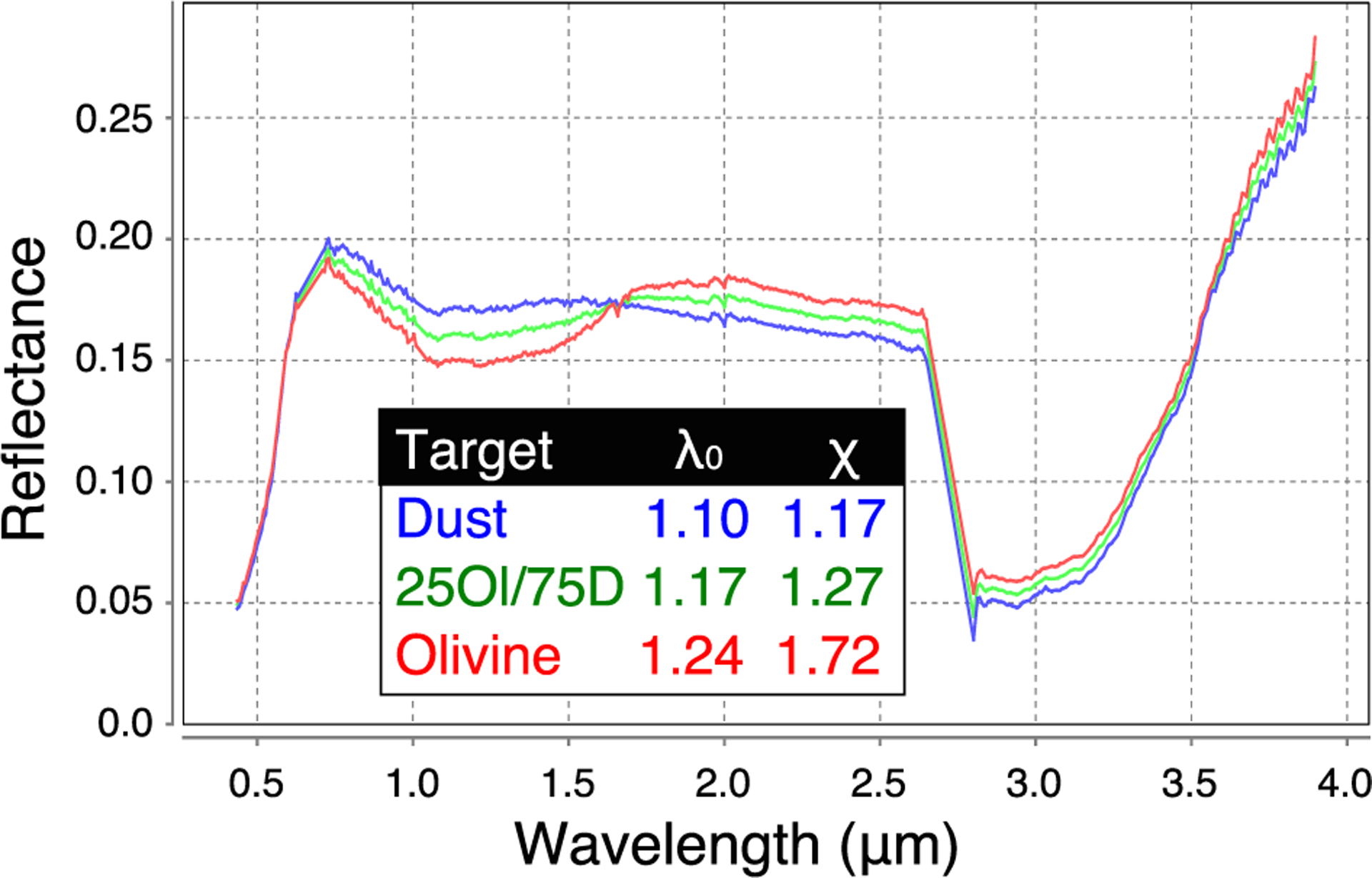
Spectral effects of mixing an example CRISM olivine spectrum with a “dusty” spectrum from the same scene in a 25% olivine/75% dust linear mixture. Locations of the spectra are shown in Figure 7.

In any CRISM pixel, it is to be expected that dust or other components on the surface might mix with olivine elsewhere in the pixel. If this is done in a spatially separated or “checkerboard” style, then the mixing can be considered to be a linear mixture of the dust spectrum with olivine. In order to assess the effect of this physical situation on the olivine 1 μm band, we have used a CRISM spectrum from FRT97E2 (see Figure 7 for continuum removed spectrum and location) which displays a redshifted olivine 1 μm band and is also likely a mixture of dust and other minerals at this pixel location. We then linearly mixed this in 25%, 50%, and 75% amounts with a spectrally bland dust-like spectrum from elsewhere in the scene, at varying percentage amounts. The original spectrum and 75% dust mixing results are shown in Figure 12.

We then carried out an asymmetric Gaussian fit to assess how much the mixing would shift the 1 μm band centroid. We found that the centroid of the original redshifted olivine pixel was 1.24 μm and the centroid of the dusty spectrum is 1.10. The mixture of 75% dust gave a centroid of 1.17 μm, which is a difference of 0.07 microns.

These results demonstrate that the effect of mixing with a “dust” spectrum in the scene is to decrease the wavelength of the centroid. The strength of this effect is roughly the same as mixing with pyroxene. As for mixing with pyroxene, the effect is to move the olivine 1 μm band to shorter wavelengths.

#### 4.1.3. Effects of Mixing With Carbonate and Phyllosilicates

**Figure 13.**
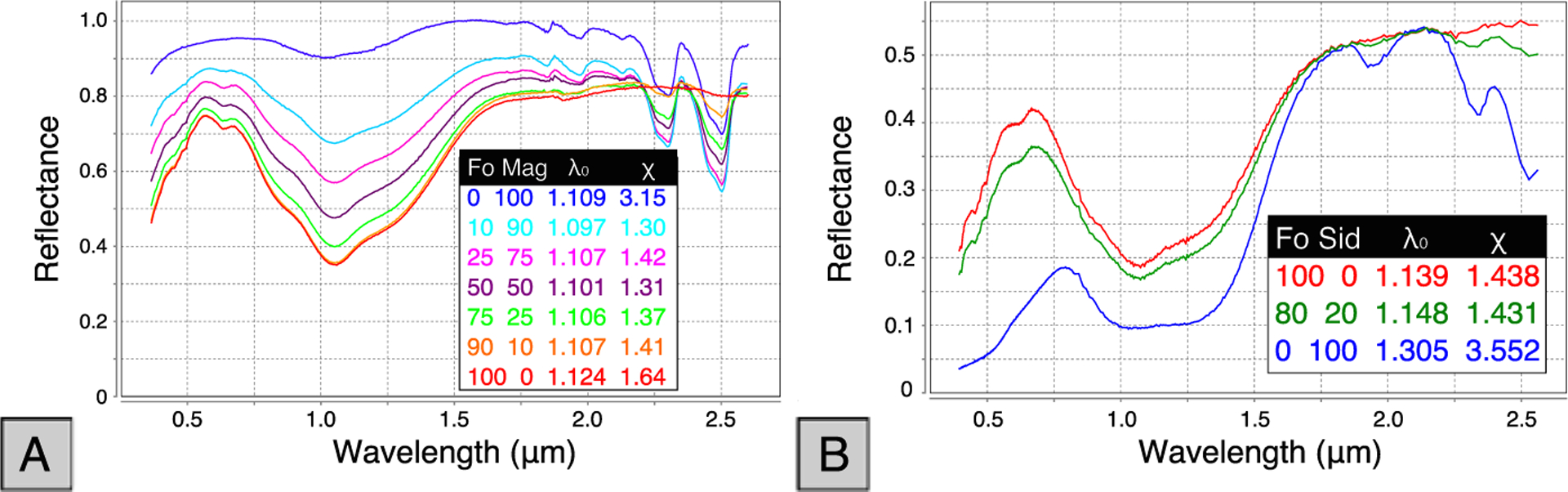
Effects of mixing olivine with carbonate on the 1 μm band position. (a) Forsterite intimately mixed with magnesite, showing the very small (10 nm) shift of the 1 μm band to shorter wavelengths with greater amounts of carbonate, data are from Bishop et al. (2013). (b) Linear mix of KI3188 Fo51 olivine with siderite HS271.3B, showing that the olivine 1 μm band is shifted by 9 nm in the case of an 80/20 mix and that the 2.3 and 2.5 μm carbonate bands are clearly visible in the mixed spectrum.

We now investigate how the presence of carbonates mixed with olivine might affect the position of the 1 μm band. In order to further assess the effect of nonlinear (intimate) mixing with carbonate, we obtained spectra from another study published in Bishop et al. (2013) and made available on RELAB. Figure 13a shows the effects of mixing forsterite with magnesite (MgCO_3_). As can be seen by comparing Figure 11 with Figure 13 a, the effect on the 1 μm band of adding magnesite to forsterite is not as profound as when pyroxene is added.

We used the asymmetric Gaussian routine to fit the 1 μm band centroid to the extremal Fo/Mag mixtures and found that the difference in centroid positions was 27 nm (between the Fo100 and Fo10/Mag90 spectra). The asymmetry difference between these two extremal mixtures was 0.34. This indicates that the effect of the magnesite mixing has about 30% of the effect of pyroxene mixing shifting the centroid of the 1 μm band. This carbonate mixing effect, while far weaker than the effect of pyroxene mixing, is still present and moves the 1 μm band in the same direction as pyroxene (to shorter wavelengths).

We now wish to assess how a carbonate with a strong 1 μm feature affects the position of the olivine 1 μm band. We could not find an intimate laboratory mixture dataset mixing siderite and olivine, so we have conducted a simple linear mixture of a siderite HS271.b from the USGS spectral library with the USGS KI3188 Fo51 olivine. Figure 13b shows the spectral effect of adding 20% carbonate and 80% olivine. First, it can be seen that there is a shift to the right for this spectrum, although it is modest (9 nm) at this 80–20 mixing level. Second, even with this small amount of siderite, the carbonate bands at 2.3 and 2.5 μm are plainly visible.

In summary, this demonstrates that siderite does have the ability to redshift the 1 μm band of olivine. However, the reason we do not believe that siderite is causing the redshift is encapsulated in Figure 8b. In that figure, we see that for the most redshifted olivines in CRISM image FRT 3E12, the carbonate 2.5 μm band not only decreases in strength but is absent from most of them. This suggests that although there may be siderite present in this CRISM scene, it is not the explanation for the most redshifted olivines we observe in FRT 3E12.

In addition, Figure 9c shows that phyllosilicate bands are absent in the most redshifted olivines in FRT3E12, as highlighted by the blue ellipse in that Figure. This indicates that phyllosilicates are also not responsible for the 1 μm band redshifts in FRT3E12. In fact, that figure shows that when phyllosilicates are present, they are mostly associated with the blueshifted olivines in the scene.

#### 4.1.4. Effect of Synthetic Olivines

**Figure 14.**
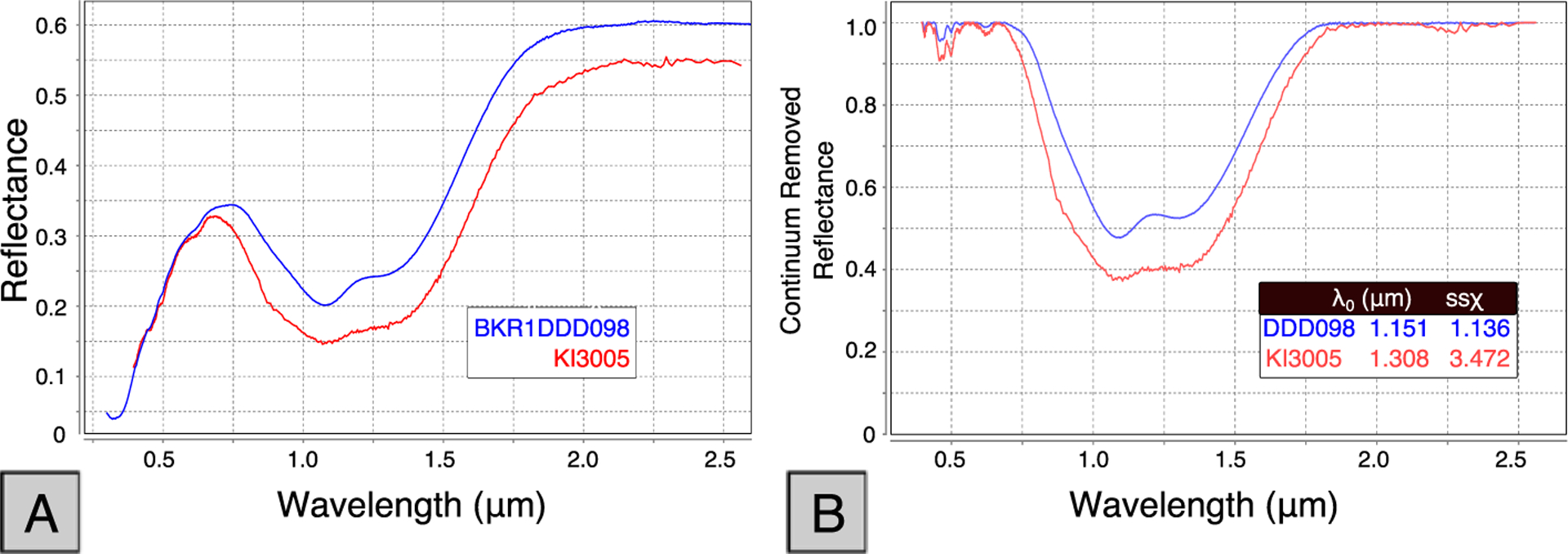
(a) Comparison of KI3005 (Fo11, <60 microns) and BKR1DDD098 (Fo0, <45 microns) spectra, showing blue shift behavior of the synthetic olivine. (b) Continuum removed spectra showing the blueshift of BKR1DDD098 with KI3005 using data from Dyar et al. (2009).

Dyar et al. (2009) reported on a suite of synthetic olivines with compositions from Fo0 to Fo90. These olivines were also discussed in an MGM study by Isaacson et al. (2014), and the spectra were then made available on RELAB. We have analyzed the spectra using the asymmetric Gaussian approach and found that the solid solution centroids were blueshifted relative to the olivines we used in this study. The Fo0-Fo90 olivines display centroids that range from 1.045 to 1.146 μm and asymmetries from 1.06 to 1.22, which is significantly less than the Kilgapait samples (compare with Figure 5). Figure 14 shows the Fo0 sample (RELAB number BKR1DDD098) compared to the Fo11 KI3005 olivine spectrum, along with a continuum removed version of both spectra, and the centroid and asymmetry best fits for both spectra.

Figure 14 clearly shows the synthetic Fo0 olivine is blue shifted to lower wavelengths relative to the Fo11 KI3005 olivine; in the scenario in Figure 14, the blue shift is 0.15 μm. We cannot explain this result at this time, and future work is required to determine the root of this mismatch. We can say, however, that this shows that the synthetic olivine spectra cannot explain the redshifting behavior we have observed in our CRISM analysis.

#### 4.1.5. Effects of Grain Size

##### 4.1.5.1. First Grain Size Study

In their seminal study of the olivine 1 μm band complex, King and Ridley (1987) reported centroid shifts of around 7 nm (0.007 μm) for Green Sand Beach (GSB: Fo89) olivine measured with 150–250 micron grain sizes compared to <60 micron size fractions. We used the asymmetric Gaussian model over the same range of grain size distributions, and for the finest grain size fraction (<60 microns), we obtained a centroid of 1.097. For the largest grain size fraction (150–200 microns), we obtained a centroid of 1.147, a difference of 50 nm, or ±25 nm.

In the same manner, we calculated an error for the asymmetry parameter based on the spread of results for the four different Green Sand Beach (GSB) olivine grain sizes. The difference in asymmetry between the largest grain size and the smallest is 1.54–1.20 = 0.34 or ±0.17. Full results are presented in the supporting information.

##### 4.1.5.2. Second Grain Size Study

In order to further determine the sensitivity of the olivine 1 μm band fitting to grain size effects, we used another dataset obtained from the RELAB database sourced from a previous study (Mustard & Pieters, 1989) that obtained spectra of a Fo92 olivine sample for a range of grain sizes. The sample numbers are BE-JFM-080 to 087.

**Figure 15.**
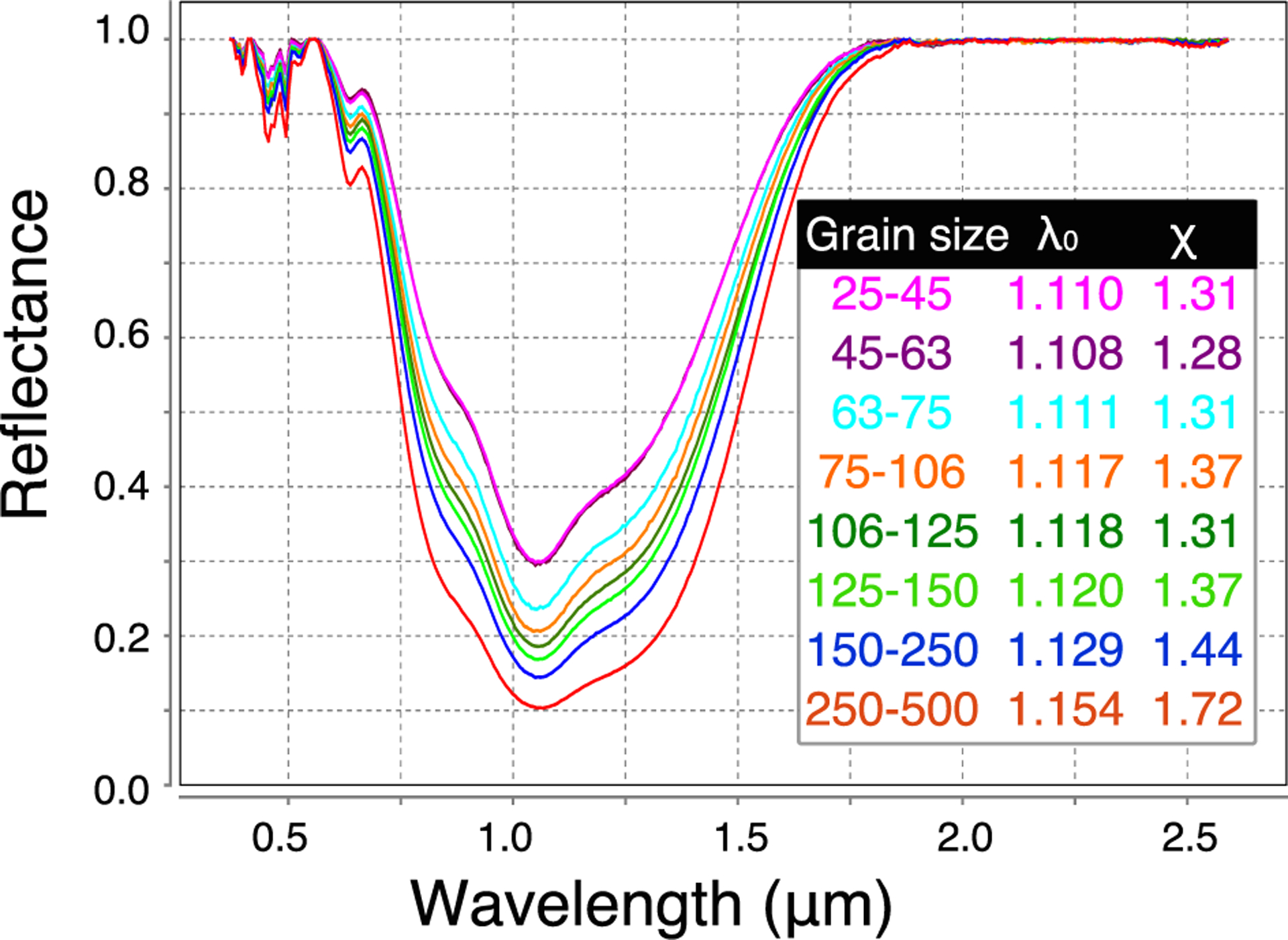
Continuum removed spectra showing the effect of grain size on the 1 μm band for the second grain size study using data from Mustard and Pieters (1989).

Figure 15 shows the continuum removed version of the spectra of eight different grain sizes of olivine from 25–45 to 250–500 microns. This range of grain sizes is twice as large as the King and Ridley Green Sand Beach grain size variations. We fit each of these spectra in an identical fashion in order to determine the variation in asymmetry and centroid. We found that the centroid of the 1 μm band was 1.110 μm for the 25–45 micron grain size and this changed to 1.154 μm for the 250–500 micron grain size, a difference of 0.044 μm. For the asymmetry, the 25–45 micron sample was 1.31, and the 250–500 micron asymmetry was 1.72. This means that the expected maximal error created by substituting a 25–45 micron sample for a 250–500 micron sample would be 0.044 μm, about the same as the error for the King and Ridley datasets in our first grain size study.

To calculate the asymmetry error, a maximal error of 0.41 is obtained between the largest and smallest datasets. This is relatively close to the asymmetry error of 0.34 calculated using the King and Ridley dataset. Since the potential error estimates were relatively close, this indicates that grain size effects from these two studies are relatively congruent. However, both these datasets are limited to grain sizes less than 500 microns.

##### 4.1.5.3. Synthetic Model Grain Size Study

The King and Ridley KI olivine dataset we carried out testing with is composed of a fine-grained material (nearly all <160 microns). As we have previously discussed, the grain size of material on Mars is not well constrained and likely quite variable over the area of the olivine-carbonate lithology; therefore, in order to explore the full range of spectral effects of variable grain size, we have chosen to synthetically model the effects of variable grain size on the apparent band position, over the range of 70 microns to 1 mm, in order to cover a wider range of grain sizes than are available in the original King and Ridley dataset.

Shkuratov et al. (1999) proposed a technique for studying the effect of grain size on the 1 μm band shape of olivine using their radiative transfer approximation. This involves using their inversion equation to estimate the imaginary index of refraction and then using this index to estimate the reflectance for differing grain sizes (see in particular their Figure 11).

In this study, we have used the same approach to estimate the imaginary index of the range of olivine spectra and then fitted the resultant reflectance spectra to estimate the variations in asymmetry and centroid for a range of grain sizes and for the varying Fo# in our olivine laboratory spectra (see supporting information for example generated spectra).

**Figure 16.**
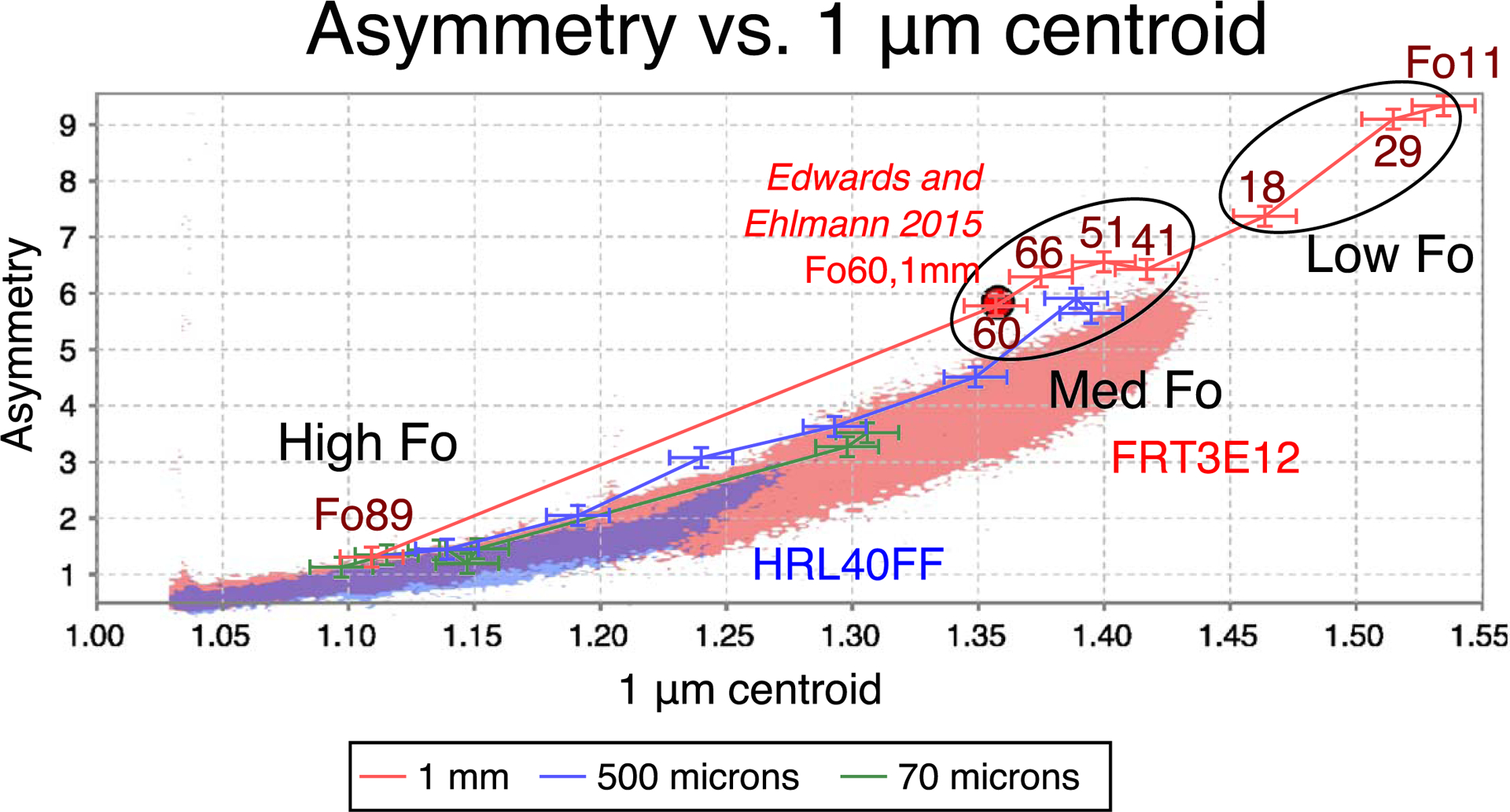
Fit of centroid versus asymmetry and olivine composition from asymmetric Gaussian fits of the olivine 1 μm band assuming a grain size of 70, 500, and 1,000 microns. Red shaded region corresponds to points in FRT3E12, blue to HRL40FF.

Figure 16 plots the relationship between the asymmetry and the 1 μm centroid position for the olivine 1 μm band, for different Fo# of olivine spectra from King and Ridley (1987). The colored lines correspond to three different average grain sizes: 70 microns, 500 microns, and 1 mm. The Fo# points are labeled on the red line only, and they are transferrable to the blue and green lines on the marked points. This shows the effect of variable grain size and composition on the olivine 1 μm band. We have also plotted the results of our asymmetric Gaussian fit for CRISM FRT 3E12 (red shading) and HRL40FF (blue outline) from Figures 8 and 6, respectively.

Figure 16 shows that there is a significant variability in the analysis model, particularly for the 1 mm grain sizes, which are attempting to fit increasingly saturated 1 μm features. The figure shows one outlier (KI3377 Fo29), which, although it plots close to the other olivines of similar Fo#, is closer to Fo11 than Fo18. It should be noted that we have not observed any CRISM spectra that plot in this range-there are no points from 3E12 that reach out this far, again suggesting that 1 mm Fo29 olivines are not present at these locations. Additionally, Figure 16 shows that two medium Fo olivine (Fo60 and 66) have interchanged positions.

Due to these two discrepancies, we have decided to group the olivines into three different groups that we can be confident we can distinguish even when the bands are saturated by 1 mm grain sizes. As shown on the plot, these groups are Low (<40), Med (40–66), and High (>66) Fo.

Known inherent errors in the asymmetric Gaussian method in Figure 16 (as discussed in section 4.1.5.1) are approximately ±25 nm in the centroid and ±0.17 in the asymmetry and are primarily due to uncertainties introduced by the reliance on experimental data that introduces spread in the process.

###### Bounds on composition and grain size.

Figure 16 gives us a way to estimate the bounds of the composition and grain size for the two CRISM images (these two are typical examples, see supporting information for more plots of this type for other images in the watershed). All of the bounds discussed below relate to the spectrally dominant olivine, and the caveats of section 4.1 should be taken into account.

We have noted the position on the plot of the recent study of Edwards and Ehlmann (2015) which carried out a Hapke fit to a CRISM spectrum in Nili Fossae (FRTC968), north of our study area, but still in the same olivine-carbonate lithological unit. They reported that an olivine of 1 mm grain size and Fo60 was required to fit the olivine 1 μm band adequately. Noting that their Hapke method is likely to differ from our method in a somewhat controlled sense, we plot their point result in Figure 16 and note two ways this plot allows us to constrain composition and grain size of the olivine:

###### 1. Composition bounds.

The Edwards and Ehlmann spot measurement is reasonable for the location from which it was taken (it corresponds to an upper part of the red shading range of FRT3E12), however is not indicative of the full range of olivine composition for the olivine-carbonate lithology. This can be seen in Figure 16 because points from FRT3E12 (red shaded in Figure 16) have a considerable tail that extends to the right of the Fo60 estimate. This indicates that although the Fo60 estimate might be a reasonable fit to the spectrum they chose (their Figure 2b), this same spectrum could also be fitted with a smaller grain size olivine with lower Fo#. The closest point on the blue line beneath the red circle corresponds to another reasonable fit using olivine of Fo18 with 500 micron grain size, for example. This is why single point assessments are not prudent in this situation, and instead, an effort to place bounds is advisable.

The points plotting in the long wavelength tail in FRT3E12 corresponds to a centroid of ~1.43 μm, which intersects the red 1 mm grain size line just to the right of Fo41. Therefore, assuming a grain size of 1 mm allows us to place a lower bound of Fo40 on the composition of the olivine in FRT3E12, where it is best exposed. It should be emphasized that this is an apparent lower band and the Fo# could be lower if some dust is present. The Fo# could also be lower if the grain size is smaller than 1 mm, as discussed above and as seen from the plot.

As discussed above, we are confident in grouping the olivines into three groups in our plot: low, medium, and high olivine. Therefore, we suggest that if we assume the grain size to be 1 mm, the composition of the olivine at Nili Fossae is likely medium Fo (40–66).

It is worth noting that Figure 16 does not give us a way to bound the upper Fo#, because as discussed above, the effects of mixing with dust, pyroxene, and carbonate will all independently shift the band position to the shorter wavelengths. This is also why we concentrate on the most extreme redshifted olivine spectra, because their redshift to longer wavelengths can only be accomplished through grain size and composition.

###### 2. Grain size bounds.

Figure 16 also gives us a way to place an upper and lower limit on the size of the spectroscopically dominant olivine grains in FRT3E12. We place the maximum and minimum values in two different ways.

First, the maximal value can be established by noting that in Figure 16, the red line showing the 1 mm grain size family is always higher than the top values of the points for FRT3E12 (the red shaded area). FRT3E12 is the most extreme endmember image that we have analyzed to date. This strongly indicates that 1 mm grain sizes are the maximum required to explain our observations.

Second, the minimal value can be established by looking at the combined range of asymmetry and centroid values for FRT3E12. The blue line corresponding to the 500 micron grain size calculations almost runs the entire length of the observations of 3E12. This means that a lower limit of 500 microns is required to fit the range of asymmetry/centroid observations. Lower grain sizes (e.g., the green 70 micron curve) cannot explain our observations in FRT3E12. Any average olivine grain size between 500 microns and 1 mm can explain our range of observations. The lower limit can be considered stronger because lower grain sizes, mixing with dust or other components, cannot explain this asymmetry/centroid range of 3E12. This does not exclude lower grain sizes in other images and locations. Higher grain sizes might be possible if there is significant dust cover in 3E12; however, we have chosen this image for its maximal range of centroid position which also suggests low amounts of dust on the outcrop in this image. Figure 8c shows an overlapping image with more dust (FRTB438) and smaller centroid range and is consistent with this conclusion.

Incidentally, this grain size bounds brings us close to the grain size range used by Koeppen and Hamilton (2008) in their thermal infrared study (710–1,000 microns).

It should be noted that Figure 16 shows that the relationship of the asymmetric Gaussian parameter set to olivine composition is not linear. Using this scheme, it is easier to determine the composition of Fe-olivine rather than Mg-olivine. Therefore, the determinations of more Fe-rich compositions can be expected to be more accurate.

### 4.2. Summary of Potential Effects on Olivine 1 μm Band

**Figure 17.**
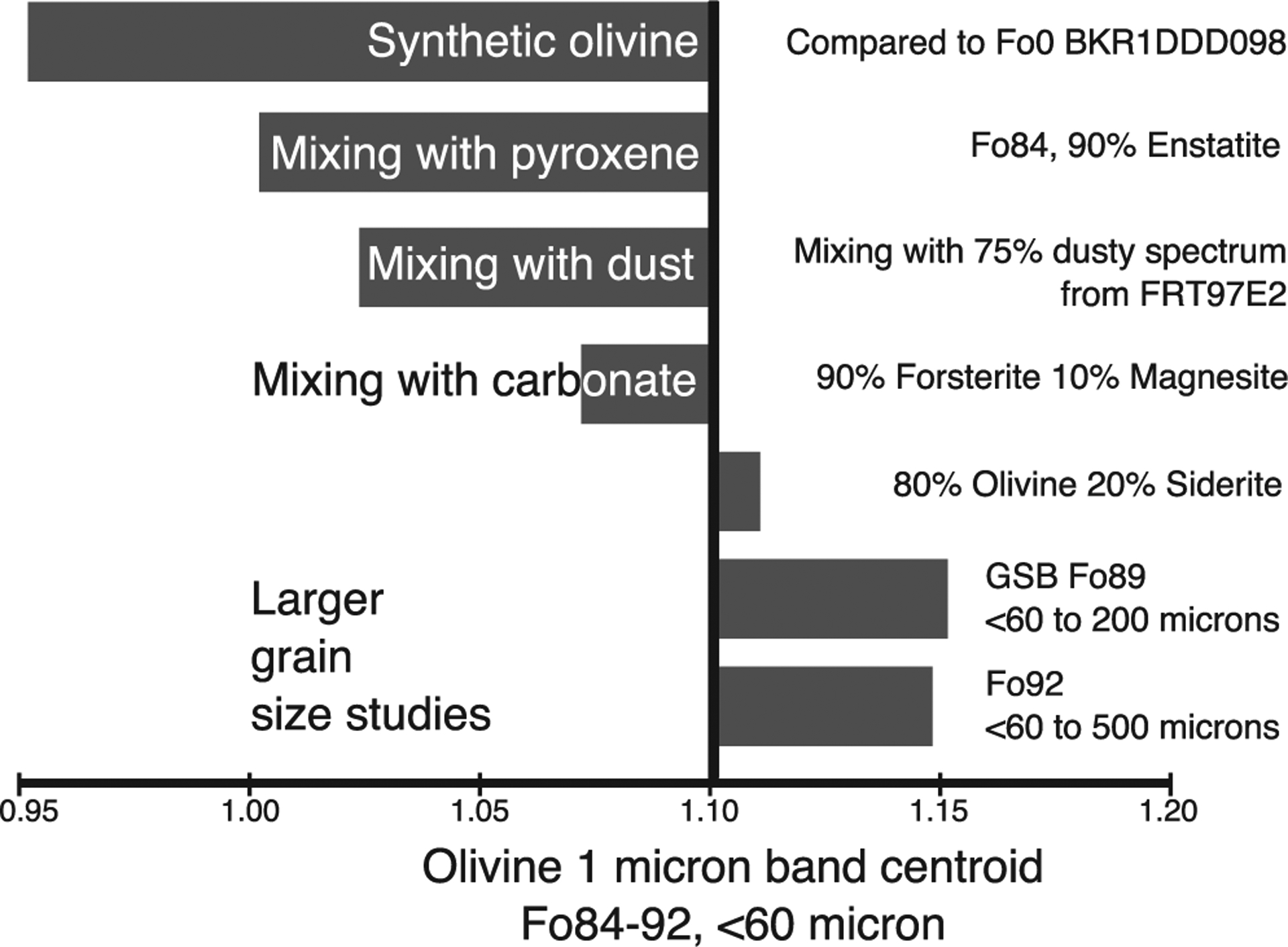
Summary of the effects of mixing and grain size on the 1 μm absorption band.

Figure 17 presents a summary of the findings of our analysis of physical effects on the olivine 1 μm band. Only composition, siderite, and larger grain sizes shift the centroid to the right. We note that while other mineral phases (including pyroxene and dust as we have shown here) are no doubt present in every CRISM olivine-bearing scene, when mixed with olivine, they all tend to shift the olivine band to shorter wavelengths, making olivine spectra appear more Mg-rich. We have identified that siderite is able to redshift the 1 μm band; however, we do not see 2.3 and 2.5 μm carbonate bands in our most redshifted olivines in CRISM FRT 3E12 (Figure 8b), which strongly indicates that siderite is not the cause of the redshift we have reported here.

### 4.3. Implications for Grain Size Effects-The Thermal Inertia Problem

Laboratory studies have demonstrated that larger grain sizes of olivine will redshift the 1 μm band to longer wavelengths, as shown here in Figure 17 and discussed by previous workers (Buz & Ehlmann, 2014; King & Ridley, 1987; Mustard et al., 2005; Poulet et al., 2007). In attempting to constrain how this process manifests itself vis-à-vis the CRISM dataset, we have looked for evidence of large grain sizes in THEMIS thermal emission datasets, which are at just slightly poorer spatial resolution than CRISM (Fergason et al., 2006). The CRISM and THEMIS derived thermal inertia maps we have discussed herein show that there is no consistent correlation between fine-grained materials and short 1 μm band centroid positions (see Figure 10). We call this the “thermal inertia problem.”

#### 4.3.1. Summary of Thermal Infrared Correlations With Olivine 1 μm Band

We firstly summarize our key observations regarding thermal inertia and the olivine 1 μm band and then discuss two possible contributing factors that could be further explored in future studies, particularly work in situ on the olivine-carbonate lithology.

We summarize the key observations of this correlation thus:

##### 1. Dune material.

In Figure 10, at the point indicated by a red arrow, a region covered by dunes visible at HiRISE resolution displays a low thermal inertia (and interpreted low effective grain size). This dune unit also shows a strong redshift in the 1 μm band. The unit appears to consist of aeolian sand dunes created from local materials, and although the grain size has likely decreased somewhat relative to surrounding bedrock (in white in Figure 10b) due to physical abrasion, the 1 μm band centroid remains significantly redshifted, as shown by the red color on Figure 10b. Even if the source of the material is not determined, the fact that fine-grained dune material here corresponds to redshifted olivine cannot be explained as a grain size effect but rather is more simply explained as a compositional effect.

##### 2. Rock competence.

Geomorphologically, in many scenes, including Figures 7 and 8, the olivine-carbonate unit appears to be less competent and more prone to breakdown than surrounding geologic units and appears to flow down slope and cover other material below it. This suggests that it is generally more friable and easy to erode than the unit below it (the olivine-phyllosilicate-bearing basement unit in Figure 8).

#### 4.3.2. Potential Models for Lack of Correlation

##### 1. Mineralogical sorting in an erosive aeolian regime.

It is not completely clear from our observations; however, we consider it likely that the aeolian dunes in Figure 10 are in fact sourced from surrounding bedrock (colored white in Figure 10b). The olivine-carbonate unit does not appear to shed boulders and appears less resistant to aeolian erosion than surrounding lithologies (such as the olivine-phyllosilicate basement unit or the mafic capping unit of the Syrtis Major lavas) (Rogers et al., 2018). When exposed, it is prone to break down, and it is easily turned into dune materials; these materials were termed “olivine-bearing dunes” by Edwards and Ehlmann (2015). An important observation is that the olivine-bearing dunes show no evidence for carbonate spectral signatures as compared to the surrounding carbonate- and olivine-bearing bedrock (see red spectrum in Figure 8a). We suggest that the current aeolian erosive regime is therefore wearing away more friable carbonate material, perhaps to create fine-grained dust (e.g., Bandfield et al., 2003) and leaving intact large grains of more resistive olivine grains in a physical weathering mineralogical sorting process. If the grain size of the olivines are slightly decreased as a result of the physical weathering, this would match the slight decrease in redshift from white outcrops in Figure 10b to red in the channel regions marked by red and green arrows.

This process is consistent with proposed mineralogical fractionation of sands in Valles Marineris (Chojnacki et al., 2013) and may be a signature of density sorting, given that olivine is denser than carbonates and will be enriched in lag deposits (Fedo et al., 2015). The process is likely to be dependent on local aeolian forcings and grain cementation, and investigating whether this process is dominant in any particular aeolian bedform is well-suited to in situ investigation with a rover.

##### 2. Thermal infrared skin depth.

Another contributing factor to the observed separation between large grain olivine features and thermal infrared measurements is that the THEMIS infrared wavelengths and CRISM visible near-infrared wavelengths have different penetration depths within a rock, which makes them sensitive to different sampling volumes within the rock. It is therefore possible that the variations of the grain size of the olivine is not dominating the thermal inertia measurements (i.e., changes in temperature). This could occur if the spectroscopically dominant olivine is in fact a volumetrically small component of the rock under study. In the aeolian weathering scenario we have outlined above, where olivine is more resistant to aeolian weathering than other grains, the majority of the aeolian lag is sourced from the non-olivine grains. This non-olivine lag will build up over time as a fine-grained cover and control the temperature of the outcrop. The large olivine grains will thereby not strongly control the temperature regime of the outcrop, even though they are detectable by CRISM.

## 5. Discussion

Thus far in this study, we have used the position and shape of the olivine 1 μm band to place bounds (~Fo40–66) on the composition and a grain size of 0.5–1 mm where it is best exposed in the olivine-carbonate lithology (section 4.1.5.3). In support of this large grain size derivation, we have demonstrated that saturation of the olivine 1 μm band occurs in some regions, and there is variability of this saturation throughout the olivine-carbonate lithology (section 3.3).

In our three key region study areas (Figures 6, 7, and 8), we have demonstrated that carbonate is associated with redshifted olivines in the centroid range of 1.2–1.3 μm (Figures 6b, 7b, and 8b). We have shown that the most saturated olivine signatures do not correlate with carbonates (Figure 8b), or with phyllosilicates (Figure 9c), *potentially* indicating that these locations are the least reacted olivines in the lithology and an extensive alteration event has not affected them.

We now discuss three key science questions that have arisen from the results of this study of the olivine-carbonate lithology.

### 5.1. Composition of Olivine Relative to Previous Results

The global studies of Poulet et al. (2007), Ody et al. (2013), and Clenet et al. (2013) found (Type 2) Fe-olivine was mostly found in a restricted region at Nili Fossae. Our results suggest that the composition of the Fe-olivine is actually medium Fo# (Fo40–66). This range overlaps with previous studies that reported estimated compositions of Nili Fossae olivine from Hoefen et al. (2003), Edwards and Ehlmann (2015), and overlaps with the low Fo# range of Koeppen & Hamilton, 2008). It overlaps with the high Fo# ranges of Skok et al. (2012) and Clenet et al. (2013). It is considerably lower in Fo# than the THEMIS study of Hamilton and Christensen (2005). We believe that the agreement with many previous estimates gives confidence that our results are largely in keeping with the previous literature. The unit is likely to experience a natural compositional range, and it is important to understand whether these variations are controlled by the original deposition and/or altered by chemical weathering and density sorting (section 4.3.2).

The composition of the Martian average mantle and crust (excluding the core) has been estimated to be ~Fo77 from SNC meteorite compositional abundances (Dreibus & Wänke, 1985) compared to the terrestrial mantle which is estimated to be ~Fo89 (Wänke, 1981). If the origin of the observed olivine-carbonate lithology is volcanic (with some later in situ aeolian modification by density sorting; Fedo et al., 2015), the original variation in Mg-Fe geochemistry of Nili Fossae olivines could have been (a) controlled by ponding and fractionation in a magma chamber isolated from the mantle (Filiberto & Dasgupta, 2011) and/or (2) reflect cooling and less vigorously mixed Martian mantle (Filiberto & Dasgupta, 2015). The relatively low temperature magmas at Nili Fossae may be driven by local crustal thinning due to the Isidis impact (Kiefer, 2005). In situ analyses of these units will shed light on these hypotheses.

### 5.2. Why Is There Residual Unaltered Olivine?

The finding of this study that the most redshifted olivine is not associated with carbonate hints at a complex relationship of what has been referred to as the olivine-carbonate lithology. We will now discuss potential carbonate formation and alteration models and address the question of why there is residual unaltered olivine present today. We will split our discussion of this question into two types of carbonatization mechanisms associated with olivine emplacement: (a) contemporaneous emplacement and (b) a distinct carbonatization event following emplacement.

#### 5.2.1. Contemporaneous Carbonatization Event

If the origin of the olivine-carbonate lithology is igneous, it is conceivable that volcanic fluids bearing CO_2_ from the mantle (Grott et al., 2011) were erupted with the olivine and were part of the material that subsequently led to the formation of carbonate-rich veining within the erupted lava body. The source of the fluids would have been connate or juvenile water sourced from a CO_2_-rich region of the mantle. The heat of the lava would have driven hydrothermal serpentinization reactions (typically <400 °C) and would have cooled relatively quickly in order to preserve the original volcanic or pyroclastic olivine clasts (Klein & Garrido, 2011; Klein & McCollom, 2013). This process may also have happened as part of an ash fall event, as has been reported in smaller terrestrial systems (Willcox et al., 2015), who documented a pyroclastic deposit containing calcite, olivine, and what they termed “- serpentine-X.”

In the igneous contemporaneous scenario, the partial carbonatization of the olivine lithology might be explained by the restriction of CO_2_-rich fluids to variable porosity or fractures in the rock that restricted fluid pathways, resulting in an uneven distribution of carbonate throughout the unit. The fact that olivine is still present in these rocks is evidence that of the relatively feeble or short-lived nature of the hydrothermal system that drove the serpentinization. In more persistent and pervasive terrestrial serpentinization systems, particularly in submarine environments, it is common for the olivine to be completely replaced by phyllosilicates.

##### Estimation of amount of CO_2_ required for contemporaneous alteration and importance of Noachian age.

Edwards and Ehlmann (2015) calculated the amount of CO_2_ required to form the Nili Fossae carbonates as the equivalent of 0.25 mbar ${\text{P}}_{{\text{CO}}_{2}}$. Grott et al. (2011) estimated that ~240 mbar of CO_2_ was outgassed during the period 4.1–3.7 Ga and at least 19 mbar in the 3.7–2 Ga period (their Table 2). This exceeds the amount required by Edwards and Ehlmann by a factor of around 100, or 10–100 times more if transport through fractures and veining occurred. While this is not a proof of this model, it strongly suggests that enough CO_2_ is likely to be available in the mantle during the 3.7–2 Ga period of interest, should the global circumstances allow it. Most importantly, this discussion shows the crucial importance of visiting Jezero crater-Mars2020 can test these CO_2_ outgassing models because the olivine-carbonate lithology was emplaced in the critical time that the CO_2_ was decreasing (Figure 2).

#### 5.2.2. Post-Emplacement Carbonatization Event

Scenarios of secondary carbonate emplacement following deposition of the olivine-rich layers are also possible. If the atmosphere was thicker when the olivine layers were deposited, it is possible that the carbonate may have been emplaced under a greenhouse atmosphere (Pollack et al., 1987), and the olivine-carbonate lithology may thus be a record of an early Martian carbon cycle. In this scenario, one might consider the variable carbonatization problematic, unless it is due to variable exposure of the olivine-carbonate lithology. The variability may occur due to CO_2_-rich fluids traveling downward in veins that connect the atmosphere with the subsurface (van Berk & Fu, 2011).

It is also possible that the carbonate was emplaced during the period when a lake was present at Jezero crater (Horgan & Anderson, 2018). The presence of similar olivine-carbonate lithologies in the Jezero crater (seen in Figure 6) and watershed (seen in Figure 7) is problematic for this scenario; although if it can be shown that the crater rim carbonates (Horgan et al., 2019) have a separate origin to the olivine-carbonate lithology at Jezero, this scenario would be more likely.

### 5.3. Relationship Between Carbonates and Mg/Fe-Phyllosilicates

This study has not concentrated on the origins of the phyllosilicates or their compositions, although we have taken them into consideration as potential contributors to the redshift of the olivine 1 μm band (see Figure 9). In situ investigation of the mineralogy associated with the carbonate layer, including the identification of specific Mg/Fe-phyllosilicates such as smectite (Bishop et al., 2008), chlorite (Viviano et al., 2013), saponite (Ehlmann et al., 2009), serpentine (Amador et al., 2018; Brown et al., 2010; Ehlmann et al., 2009), and/or talc (Brown et al., 2010; Viviano et al., 2013), will help reveal the alteration conditions and temperature and pressure conditions that accompanied the hydration event and may determine whether it was associated with a serpentinization, sedimentary, or leaching process. The formation conditions are crucial because the presence of talc-carbonate resulting from the carbonatization of serpentine has been examined in Earth analogs in terrestrial greenstone belts such as the Pilbara in Western Australia (Brown et al., 2005, 2006), where talc-bearing komatiite cumulate units of the Mt Ada Basalt underlie the siliceous, stromatolite-bearing Strelley Pool Chert unit (Van Kranendonk et al., 2008). An in situ investigation of the Mg/Fe-phyllosilicate mineralogy and the nature of the hydration event is therefore a critical task in understanding the astrobiological potential of the carbonate and phyllosilicate deposits at Nili Fossae.

### 5.4. Proposed Olivine-Carbonate Lithology Formation Scenarios

Table 1 presents a summary of potential formation scenarios we are presently aware of that may have played a role in the emplacement and/or alteration of the olivine-carbonate lithology in the Jezero crater region. Each of the formation and alteration scenarios in Table 1 present a plausible formation mechanism for the emplacement of carbonate at Nili Fossae and potentially the accompanying olivine and associated Mg/Fe-phyllosilicates.

In constructing Table 1, we have assessed each physical scenario and its ability to account for our three primary observations. If the scenario would not be expected to produce the specific observations, no ticks are assigned. If the scenario has not been documented to produce a similar observation, but we assess that it might, one tick is assigned. If the scenario has been documented to result in the observation, two ticks are assigned. If the scenario was specifically designed or is fully capable of account for the observation, three ticks are assigned. In cases of scenarios where terrestrial observations might not match exactly (e.g., carbonate types in dry lake environments) but could easily translate to match the observations of carbonate types on Mars, two ticks are assigned.

We have thus made a qualitative assessment of the ability of each scenario to address the findings of this study, based on current scenarios in the literature. At this time, we do not have the required data to conclude which scenario is correct and/or whether multiple scenarios were at play. Multiple scenarios may combine to account for missing observations; for example, the combination of an ash fall deposit and later subsurface alteration may combine to give a full complement of ticks. In the last column of the table, we have provided testable hypotheses and/or observables for each scenario that could be used to evaluate each mechanism with future in situ exploration.

## 6. Conclusions

This study has provided four new insights into the Nili Fossae olivine-carbonate lithology that may be used to test current and future formation models.

1. We have established that within the olivine-carbonate lithology, there is band saturation of the olivine 1 μm band complex. Variability of this saturation effect has enabled us to place limits on the grain size of olivine controlling the band shape. We have shown using an asymmetric Gaussian fit in three key regions of Nili Fossae that the centroid and asymmetry that the presence of at least 500 microns grain size olivine is required, and at most 1 mm grains can be accommodated. Additionally, if the 1 mm grain size is assumed, we have shown evidence that these can be fitted with a Fo40–66 olivine.
2. We have established that the portion of the Nili Fossae olivine characterized in the VNIR as Fe-olivine (or large grain size material) by Mustard et al. (2005), Poulet et al. (2007), mapped as Type II olivine by Ody et al. (2013) and mapped by Clenet et al. (2013), is in fact spatially associated with the carbonates discovered at Nili Fossae by Ehlmann, Mustard, Murchie, et al. (2008). We have established that this association is only with intermediate saturation olivine units (centroid 1.2–1.3 μm) and not with maximally or minimally saturated olivines.
3. We have studied thermal inertia maps from THEMIS to better characterize the olivine-carbonate lithology. We have showed that the olivine-carbonate 1 μm centroid does not display a reliable correlation with thermal inertia, and we have posited several potential reasons for this.
4. We have demonstrated that obscuration by dust and other components can shift the olivine band to shorter wavelengths; however, it cannot redshift the band or explain our observations of redshift and band saturation. We have demonstrated that the absence of 2.3 and 2.5 μm bands associated with the most redshifted 1 μm bands means that phyllosilicates and carbonates cannot be responsible for the observed extremal redshifts.
5. We have demonstrated that the observations we have made of olivine band saturation and centroid requires a combination of large grain size and medium Fo composition. We fully realize our calculations, and the non-linear curves we have derived are dependent on a particular and relatively simple radiative transfer model; however, there are good physical reasons to believe that these large grain size-saturation trends will hold. For example, band saturation occurs for large grain-sized water ice in the summer exposures of the north polar residual ice cap (Langevin et al., 2005).

This study has therefore strengthened our geological understanding of two previously heavily studied but heretofore unlinked key units of the Nili Fossae region. It has placed constraints on the grain size (>500 microns) and composition (Fo40–66) of the olivine-carbonate lithology using the centroid position and asymmetry of the 1 μm olivine band complex. In addition, we have discussed several olivine-carbonate formation pathways and how this correlation between olivine and carbonate is expected to impact previously proposed formation scenarios of the Nili Fossae olivine-carbonate lithology.

## Supplementary Material

Supporting Material

## Figures and Tables

**Table 1:** Possible Formation Scenarios, Science Questions Arising From This Study, and Testable Predictions for Jezero Olivine-Carbonate Lithology

Scenarios	Observations			In situ testable hypothesis or observable
	1. Olivine Emplacement/composition/grain size	2. Partial carbonatization	3. Associated Mg/Fe-phyllo	
Volcanic succession on early Martian crust (Hamilton & Christensen, 2005; Tornabene et al., 2008), by dyke driven volcanism (Bramble et al., 2017) with accompanying deuteric alteration; serpentinization reactions driven by heat of volcanic emplacement (Brown et al., 2010; Viviano et al., 2013)	√√√	√√√	√√√	Lava flow units in stratigraphic section, potentially cumulate clast textures and large grain sizes
Impact-driven hydrothermal activity by a melt sheet (Hoefen et al., 2003; Mustard et al., 2007, 2009) serpentinization reactions driven by hydrothermal activity from heat of impact (Osinski et al., 2013)	√√	√√√	√√	Superposed impact melt sheet
Emplacement by olivine-rich pyroclastic ash flow at low temperature (Kremer et al., 2019; Mandon et al. 2019)	√√√		√	Pyroclastic ash unit in stratigraphic section, small grain sizes
Subsurface alteration under thicker CO_2_ atmosphere; serpentinization reactions driven by diagenesis and upper crustal hydrothermal processes (Edwards & Ehlmann, 2015; van Berk & Fu, 2011)		√√√	√√√	Indicators of subsurface alteration in carbonate and zoning (van Berk & Fu, 2011)
Hydrothermal alteration in thermal springs environment (Walter & Des Marais, 1993) or alteration of volcanic tephra by ephemeral waters (Ruff et al., 2014)		√√√	√√	Mineralogical and physical evidence of tephra-like deposits showing hydrothermal alteration
Deep subsurface reservoir of carbonate exposed by meteor impact (Glotch & Rogers, 2013; Michalski & Niles, 2010)		√√√	√√	Layering, exposure in deep crater walls or peaks
Cold ophiolite-hosted serpentinization, as in the terrestrial analogs in California (Campbell et al., 2002; Schulte et al., 2006) or the Oman ophiolite (Paukert et al., 2012)	√√	√√	√√	Low temperature serpentinization minerals
Low temperature leaching, as in terrestrial analog of Antarctic carbonate rinds (Doran et al., 1998)		√√	√	Carbonate in surface rinds
Precipitation of carbonate directly into shoreline of shallow lake (Horgan & Anderson, 2018) or dry lake (Baldridge et al., 2009; Marion et al., 2009) or marine basin, includes scenarios of Noachian Martian ocean preserved at Nili Fossae (Russell et al., 2014)		√	√√	Marginal carbonates, Carbonate reefs, microbiolites, stromatolites exhalative/smoker structures
Hydrothermal formation of carbonates and clays from an olivine-bearing Martian basalt under a thick CO_2_ atmosphere (Dehouck et al., 2014; Pollack et al., 1987) or under a high pressure and temperature atmosphere (Cannon et al., 2017)	√√√	√√	√√√	High temperature or pressure mineral phases
Deposition of olivine/carbonate sediment in a large aeolian dune field, such as the lower unit of the Burns Formation (Grotzinger et al., 2005)	√√	√	√	Aeolian sedimentary features in the carbonate

*Note*. No ticks indicates that the scenario cannot address this question, one tick indicates that the scenario might account for this question; two ticks indicate that it partially deals with the question, and three ticks mean it specifically addresses the question. See discussion in section 5.4.

## Key Points:

- We identify a previously unrecognized relationship between carbonates in the Jezero crater region and the 1 μm band of associated olivine
- We use the shape and centroid of the 1 μm band to place bounds on the grain size (>500 microns) and composition (Fo ~40–66) of the olivine
- We examine the thermal inertia of carbonate- and olivine-bearing regions and find that it is not correlated with the 1 μm band centroid position
