# Supplementary material for: Olivine-Carbonate Mineralogy of the Jezero Crater Region
Source: J Geophys Res Planets. Author manuscript; Available in PMC 2020 Oct 28. (PMC7592698; doi:10.1029/2019je006011)
Supplement: Supporting Material [file NIHMS1624893-supplement-Supporting_Material.docx]

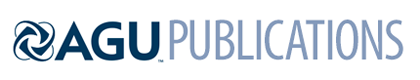


*Journal of Geophysical Research*

Supporting Information for

**Olivine-Carbonate Mineralogy of the Jezero Crater Region**

A. J. Brown^1^, C. E. Viviano^2^ and T. A. Goudge^3^

^1^Plancius Research, Severna Park, MD 21146. ^2^Johns Hopkins Applied Physics Laboratory, MD. ^3^Jackson School of Geosciences, University of Texas, TX.

**Contents of this file**

1. CRISM Observation Types

2. CRISM/HiRISE Data List

3. Asymmetric Gaussian band modeling

3.1 Step by step description of the fitting algorithm

3.2 Asymmetric Gaussian Fit Results

4. Sources of spectra for mixing analysis reported in the paper

5. Example Shkuratov model spectra

6. Carbonate comparison map

**1. CRISM Observation Types**

CRISM is a visible to near-infrared imaging spectrometer with 545 channels from 0.365-3.94 μm (Murchie et al., 2007). CRISM has short (S) and long (L) wavelength channels, the targeted products we used in this study have these two channels merged. Table S.1 lists the CRISM observation types and their characteristic properties. We have listed the approximate footprint – the non-mapping images are transformed into a bow-tie shape due to variable tilt angles on the surface. Mapping observations are ~10km wide, but vary in length.

The CRISM targeted observations presented here are Map-projected Targeted Reduced Data Records (MTRDRs), derived by processing CRISM hyperspectral targeted observations through a series of standard spectral corrections, spatial transformations, and renderings (Seelos et al., 2012). All post-processing was performed prior to the production of summary or browse products (Viviano-Beck et al., 2014), which we have used to visualize the distribution of observed phases in both targeted and mapping data.

| Observation type | Abbreviation | # of channels | Field of view | Approx. footprint |
| --- | --- | --- | --- | --- |
| Full Resolution Targeted | FRT | 545 | 18m/pixel | 10x10km |
| Full Resolution Short | FRS | 545 | 18m/pixel | 2x10km |
| Multispectral mapping | MSP | 72 | 180m/pixel | Variablex10km |
| Half Resolution Long | HRL | 545 | 36m/pixel | 20x10km |
| Half Resolution Short | HRS | 545 | 36m/pixel | 5x10km |

**Table S.1.** – CRISM observation types

2. CRISM/HiRISE Data List

Table S.2 provides a list of CRISM/HiRISE images that were used to complete our mapping for this study. The images are listed in order by the Figure in the paper that they appear in.

| CRISM ID | Central Lat, Lon | Alteration phases identified | Primary phases identified |
| --- | --- | --- | --- |
| Figure 6 | | | |
| FRT47A3 | 18.565495, 77.508885 | Mg-carb, Mg-phyllo. | Fe/Mg olivine |
| HRL40FF | 18.504645, 77.418175 | Mg-carb, Mg-phyllo | Fe/Mg olivine |
| Figure 7 | | | |
| FRT97E2 | 19.226475, 76.439725 | Mg-phyllo, Mg carb | Fe/Mg olivine |
| FRT23370 | 19.137,  76.208 | Mg-phyllo, Mg-carb | Fe/Mg olivine |
| Figure 8 | | | |
| FRT3E12 | 22.260495,  77.076525 | Mg-phyllo, Mg-carb | Fe/Mg olivine |
| FRTB438 | 22.16679,  77.16892 | Mg-phyllo, Mg-carb | Fe/Mg olivine |
| Figure 10 | | | |
| (c) ESP_026992_2025_RED, (d) PSP_002888_2025_RED | | | |

**Table S.2.** List of CRISM/HiRISE IDs analyzed in the manuscript.

3. Asymmetric Gaussian band modeling

In this section we provide the details of how the asymmetric band mapping was carried out in order to compute the apparent Fo# of the olivine spectra presented in this paper.

Figure S.1 demonstrates the method by which the olivine bands have been fit using an Asymmetric Gaussian shape. It is a copy of Figure 4 from the text which is replicated here for the benefit of the reader in understanding the algorithm. This process was first described by Brown et al. (2010) and is based on the fitting technique of Brown (2006). It is similar in many respects to the SPECPR program described by King and Ridley (1987).

**3.1 Step by step description of the fitting algorithm**

The following steps are taken to fit the olivine 1 μm band complex.

**Step 1 - Ingestion.** The olivine spectrum is ingested into the program at the original resolution. We provide an option for the user to select a high frequency smoothing routine based on the Savitzky-Golay (1964) algorithm to decrease small scale noise. The smoothing routine is designed to reduce noise from CRISM spectra of the 1 μm band. The routine we adopted also is designed to be consistent in spectral distribution across FRT and MSP images. However, we have found that it is not necessary to use the smoothing routine on the MTRDR FRT or laboratory spectra we have used in this study, therefore no smoothing is carried out on the results shown in this paper.

The code adopts the full resolution targeted spectral distribution as the basis for the fitting analysis. If the spectra are interpolated to introduce new points and then these points are linearly interpolated to match the FRT distribution. In this way, the Asymmetric Gaussian fitting routine is able to achieve very consistent results across the range of CRISM spectral resolutions it is presented with. The (minor) effects of the decrease in MSP resolution are reported further in Section 3.3 below.

**Step 2 - Continuum Removal.** The routine uses user defined shoulders to find the minimum and maximum wavelengths. In this project we used 0.75 and 1.75 μm, respectively. A straight line is drawn between these two points. A continuum is fit to this line by drawing a straight line between two shoulder points. This is used as a reference, and the band values are found as the differences of the reflectance values to this line.

In our calculation of the continuum removed spectrum, following Clark et al. (1987) we take the approach of dividing the spectrum by the line drawn between the two chosen shoulder points. We call this result the “continuum removed reflectance”. For example, if the linear interpolation is around 0.5, and the spectrum is 0.2 at this point, the continuum removed reflectance will be 0.2/0.5=0.4. For the purposes of this study, we do not fit a complete hull to the spectrum, and instead just focus on two fixed shoulder points.

**Step 3 – Gaussian Model fit.** Next, the routine automatically finds the minimum of the continuum removed band and approximates the half-width half-maximum (i.e. half the width at halfway up from the maximum band depth) for the high and low side of the band. The routine then generates an asymmetric shape to approximate the absorption band.

The equation for the asymmetric shape is:

If $\lambda\leq\lambda_{0}$ $f\left( \lambda\right)=\alpha\text{exp}\left( -\left[ \frac{\lambda-\lambda_{0}}{\text{ }\sigma^{2}} \right]^{2} \right)$ if *λ>λ_0_* $f\left( \lambda\right)=\alpha\text{exp}\left( -\left[ \frac{\lambda-\lambda_{0}}{\left( \text{}\chi\sigma\right)^{2}} \right]^{2} \right)$ (1)

The parameters are centroid *λ_0_*, amplitude α, half width half maximum (HWHM in μm) σ, and the asymmetry parameter χ. The asymmetry parameter is unbounded – values less than 1 indicate right asymmetry, values greater than 1 indicate left asymmetry.

The band minimum is used as the initial value of the centroid of the Gaussian shape, and then is treated as a free parameter. The fitting is done in 'energy' space, meaning wavenumber space, and hence the spectrum is inverted, as advocated by (Rossman, 1988). The following equation is used to convert from continuum removed reflectance to what we call “apparent absorbance”:

$f\left( \lambda\right)=-{log}_{10}\alpha\left( \lambda\right)$ (2)

This equation converts, for example, a value of 1.0 in a continuum removed spectrum to zero, and values such as 0.2 to 0.69 or 0.4 to 0.39.

All parameters are allowed to run free during the fit (except the wavelength *λ* which is stepped to match the CRISM wavelengths)*.* There are two equations in (1) that separately handle the situation when the wavelength *λ* is larger or smaller than the centroid *λ_0_*. If the wavelength is less than the centroid, the Gaussian shape for this half is a standard Gaussian shape (equation on left of (1)). If the wavelength is greater than the centroid, the Gaussian shape is altered by the insertion of the asymmetry parameter χ (equation on right of (1)).

If the asymmetry parameter is less than 1, the width of the shape is decreased (relative to the left) on the right, we call this “right asymmetric” because the centroid has effectively moved to the right. If the asymmetry parameter is greater that one, the width of the right side shape is increased (relative to the left), we call this “left asymmetric” because the centroid has effectively moved to the left – Figure S.1 shows an example of a left asymmetric fit in Step 3 in the two right hand columns.

| Parameter | Purpose |
| --- | --- |
| *λ_0_* | The centroid is the center of the Gaussian figure |
| α | The amplitude of the Gaussian shape is the height of the figure |
| σ | The half width half maximum of the Gaussian figure (in μm) |
| χ | The asymmetry parameter <1 is right asymmetric, >1 is left asymmetric |

**Table S.3.** Purpose of parameters for the Gaussian fit equation (1)

**Step 4 – Residual Calculation.** The model asymmetrical Gaussian shape is subtracted from the CRISM spectrum at each wavelength point to determine the residuals of the fit. This difference is squared to handle negative values, and the squares are summed and divided by the number of points to calculate the residual. This residual is then used to determine whether the fit has reached a sufficiently close fit, if so, the spectrum is inverted and converted back to μm, the results are reported to the user and the routine finishes. If the fit is not sufficiently small, Step 3 is repeated with an improved asymmetry and centroid position. To obtain the next solution for the fit we use a Nelder-Mead simplex algorithm, which is simple and robust (Nelder and Mead, 1965).

**Choice of residual threshold.** We provide the Nelder-Mead algorithm with a tolerance of 10^-4^ and calculate the root mean square difference divided by the number of points. In some sense, our errors as seen in Step 4 of Figure S.1 can be expected to be “poor” because we are trying to fit a more complex, 3 band solution with just one asymmetric band. We experimented with decreasing our tolerance (to 10^-9^ and 10^-15^) and our fits did not change to 3 decimal places, therefore we settled on a tolerance of 10^-4^ for speed and efficiency.

**Flow chart and extrema examples.** Figure S.1 shows a flow chart and two examples of the asymmetric band fitting operating on laboratory olivine spectra. The two samples are chosen to illustrate why this method is effective in separating the olivine composition. The GDS70.d sample has Fo#89 and the KI3005 has Fo#11, so these can be considered as extremal endmembers. In Step 1, the two original spectra show clear differences in the 1 μm band. Step 2 shows the broader band of the low Fo KI3005 sample compared to a narrower band for GDS70.d. In Step 3, the eventual fit is shown. Note that the spectra are flipped left right and inverted top to bottom in the last two rows to accomplish the “energy space” fit. For the GDS70.d spectrum, the Gaussian band is more symmetric and for the KI3005 spectrum, the fitting band is more asymmetric. The centroid positions are shown with vertical lines. Step 4 shows the residuals of each fit.


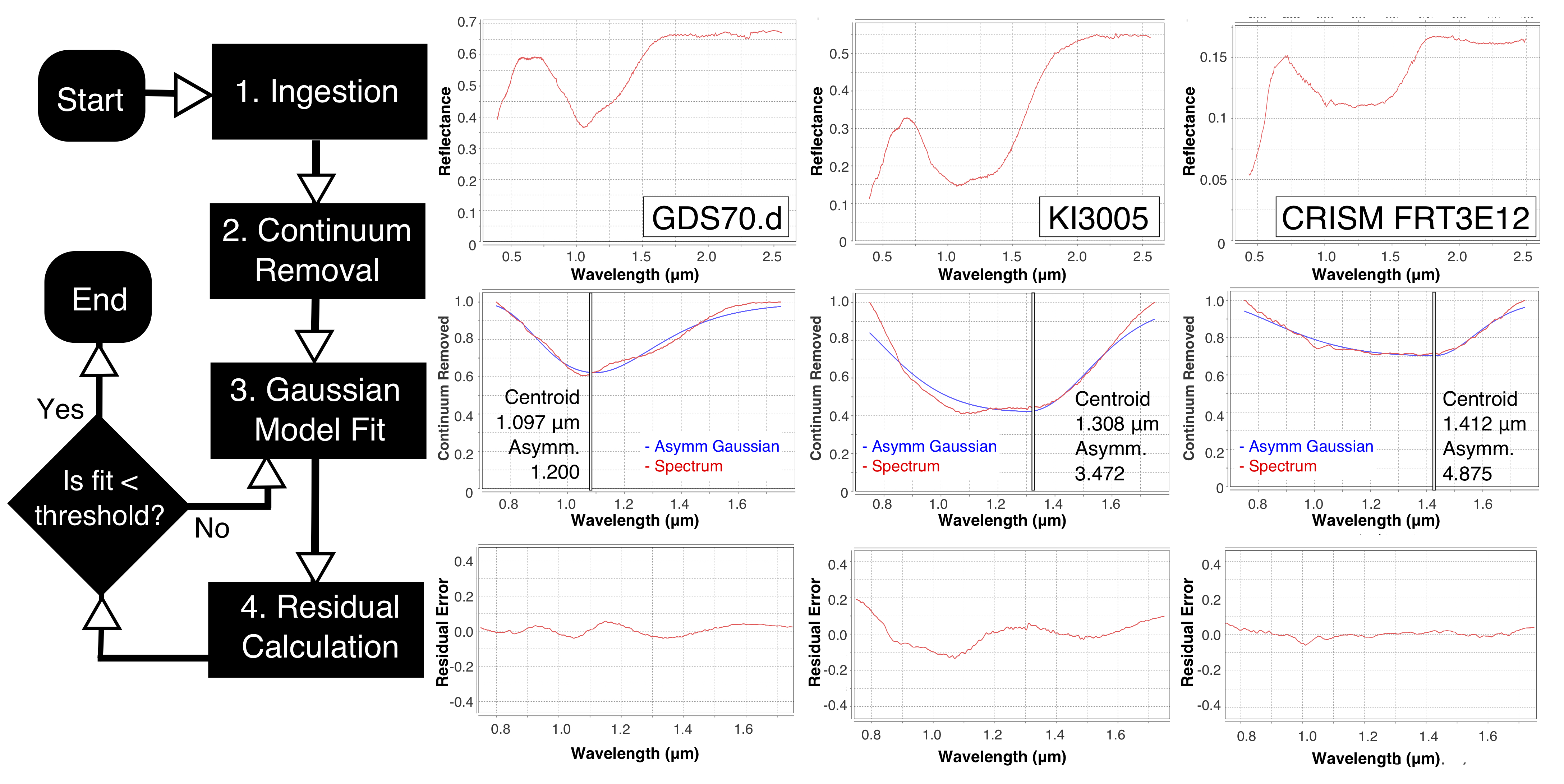


Figure S.1. Asymmetric band modeling of the library spectra of two olivine samples (GDS70.d and KI3005), and a CRISM spectrum from FRT3E12. This is a replica of Figure 4 of the text, with the addition of a flow chart for the fitting process.

**3.2 Asymmetric Gaussian Fit Results**

To prepare the plot of the asymmetry and centroid results of the 1 μm band in Figure 4, 5 and Figure 16 of the paper, we used the olivine library spectra that are listed below in Table S.4. Also included in the table are the Gaussian fit results (for asymmetry and centroid) that we obtained for each laboratory olivine spectrum.

| Name | Fo# | Grain size in microns | Asymmetry χ | Centroid μ*_0_* in μm | Band min. King +Ridley (μm) | Reference |
| --- | --- | --- | --- | --- | --- | --- |
| KI3005 | 11 | < 60 | 3.472 | 1.308 | 1.08 | King and Ridley (1987) |
| KI3377 | 18 | <60 | 3.514 | 1.306 | 1.08 | King and Ridley (1987) |
| KI3291 | 29 | < 60 | 1.979 | 1.214 | 1.08 | King and Ridley (1987) |
| KI4143 | 41 | < 60 | 1.446 | 1.151 | 1.075 | King and Ridley (1987) |
| KI3188 | 51 | < 60 | 1.439 | 1.139 | 1.07 | King and Ridley (1987) |
| KI3189 | 60 | < 60 | 1.425 | 1.137 | 1.065 | King and Ridley (1987) |
| KI3054 | 66 | < 60 | 1.339 | 1.115 | 1.06 | King and Ridley (1987) |
| GDS70.a GSB | 89 | 150-200 | 1.541 | 1.147 | 1.055 | King and Ridley (1987) |
| GDS70.b GSB | 89 | 104-150 | 1.481 | 1.137 | Not specified | King and Ridley (1987) |
| GDS70.c GSB | 89 | 60-104 | 1.360 | 1.115 | 1.048 | King and Ridley (1987) |
| GDS70.d GSB | 89 | < 60 | 1.200 | 1.097 | 1.048 | King and Ridley (1987) |
| NMNH137044.a | 92 | 160 | 1.42 | 1.119 | N/A | USGS spec lib |
| NMNH137044.b | 92 | < 74 | 1.356 | 1.111 | N/A | USGS spec lib |
| C1PO72 | 36 | < 70 | 1.578 | 1.160 | N/A | Sunshine and Pieters (1998) |
| C1PO74 | 42 | < 70 | 1.339 | 1.130 | N/A | Sunshine and Pieters (1998) |
| C3PO65 | 84 | <70 | 1.309 | 1.105 | N/A | Sunshine and Pieters (1998) |
| C1PO31 | 84 | <70 | 1.361 | 1.112 | N/A | Sunshine and Pieters (1998) |
| C1PO27 | 89 | <70 | 1.313 | 1.106 | N/A | Sunshine and Pieters (1998) |
| C3PO51 | 90 | < 70 | 1.351 | 1.110 | N/A | Sunshine and Pieters (1998) |
| C3PO65 | 92 | < 70 | 1.309 | 1.106 | N/A | Sunshine and Pieters (1998) |
| C3PO53 | 97 | < 70 | 1.359 | 1.102 | N/A | Sunshine and Pieters (1998) |

**Table S.4.** 21 Laboratory Olivine samples along with reference that reported measured Fo#, grain size, and the centroid and asymmetry results of Asymmetric Gaussian band fitting. “KI” stands for Kiglapait, “GSB” for Green Sand Beach. Centroids of King and Ridley were obtained by visual estimation from Figure 6 of their paper. Highlighted spectra have grain size ~70 microns and are plotted in Figure 5 of the manuscript.

4. Sources of spectra for mixing analysis reported in the paper

To prepare Figures 11, 13, 14 and 15, testing the effect of mixing with other components on the Asymmetric Gaussian fitting, we used a variety of spectra from different sources in the literature. These are listed in Table S.5. below.

| Name | Range in μm | Source | Reference | Figure |
| --- | --- | --- | --- | --- |
| AG-TJM-014-70-OL-30-LCP | 0.371-2.555 | RELAB | Corrigan et al. (2007) | 11 |
| AG-TJM-017-10-OL-90-LCP | 0.371-2.555 | RELAB | Corrigan et al. (2007) | 11 |
| AG-TJM-018-30-OL-70-LCP | 0.371-2.555 | RELAB | Corrigan et al. (2007) | 11 |
| AG-TJM-019-50-OL-50-LCP | 0.371-2.555 | RELAB | Corrigan et al. (2007) | 11 |
| AG-TJM-020-90-OL-10-LCP | 0.371-2.555 | RELAB | Corrigan et al. (2007) | 11 |
| JB-JLB-A15-50-Fo-50-Ens | 0.371-2.555 | RELAB | Freeman et al. (2010) | 11 |
| JB-JLB-A16-25-Fo-75-Ens | 0.371-2.555 | RELAB | Freeman et al. (2010) | 11 |
| JB-JLB-A16-75-Fo-25-Ens | 0.371-2.555 | RELAB | Freeman et al. (2010) | 11 |
| JB-JLB-945a (Forsterite) | 0.364-2.608 | RELAB | Bishop et al. (2013) | 13 |
| JB-JLB-946 (Magnesite) | 0.364-2.608 | RELAB | Bishop et al. (2013) | 13 |
| JB-JLB-954-25-Fo-75-Mag | 0.364-2.608 | RELAB | Bishop et al. (2013) | 13 |
| JB-JLB-955-50-Fo-50-Mag | 0.364-2.608 | RELAB | Bishop et al. (2013) | 13 |
| JB-JLB-956-75-Fo-25-Mag | 0.364-2.608 | RELAB | Bishop et al. (2013) | 13 |
| JB-JLB-957-90-Fo-10-Mag | 0.364-2.608 | RELAB | Bishop et al. (2013) | 13 |
| JB-JLB-958-10-Fo-90-Mag | 0.364-2.608 | RELAB | Bishop et al. (2013) | 13 |
| BKR1DDD098 | 0.3-12.835 | RELAB | Dyar et al. (2009) | 14 |
| BE-JFM-081 \| OLV-SC 25-45 um | 0.364-2.608 | RELAB | Mustard and Pieters (1989) | 15 |
| BE-JFM-082 \| OLV-SC 45-63 um | 0.364-2.608 | RELAB | Mustard and Pieters (1989) | 15 |
| BE-JFM-083 \| OLV-SC 63-75 um | 0.364-2.608 | RELAB | Mustard and Pieters (1989) | 15 |
| BE-JFM-084 \| OLV-SC 75-106 um | 0.364-2.608 | RELAB | Mustard and Pieters (1989) | 15 |
| BE-JFM-085 \| OLV-SC 106-125 um | 0.364-2.608 | RELAB | Mustard and Pieters (1989) | 15 |
| BE-JFM-086 \| OLV-SC 125-150 um | 0.364-2.608 | RELAB | Mustard and Pieters (1989) | 15 |
| BE-JFM-087 \| OLV-SC 150-250 um | 0.364-2.608 | RELAB | Mustard and Pieters (1989) | 15 |
| BE-JFM-088 \| OLV-SC 250-500 um | 0.364-2.608 | RELAB | Mustard and Pieters (1989) | 15 |

**Table S.5.** Source of spectral data for Figure 11 (LCP mixing), Figure 13 (Carbonate mixing) Figure 14 (Synthetic olivines) and Figure 15 (Grain size variation) of the text.

5. Example Shkuratov model spectra

To prepare Figure 16 in the main text, we used the Shkuratov radiative transfer model to determine the reflectance spectra for olivines of varying Fo# and grain size. Figure S.2 shows an example run of these spectra for olivine KI3189, which is Fo60. The grain sizes 70, 250, 500 and 1000 microns are shown. The largest grain size (500 microns and 1mm) can be seen to display (increasingly dominant) band saturation.


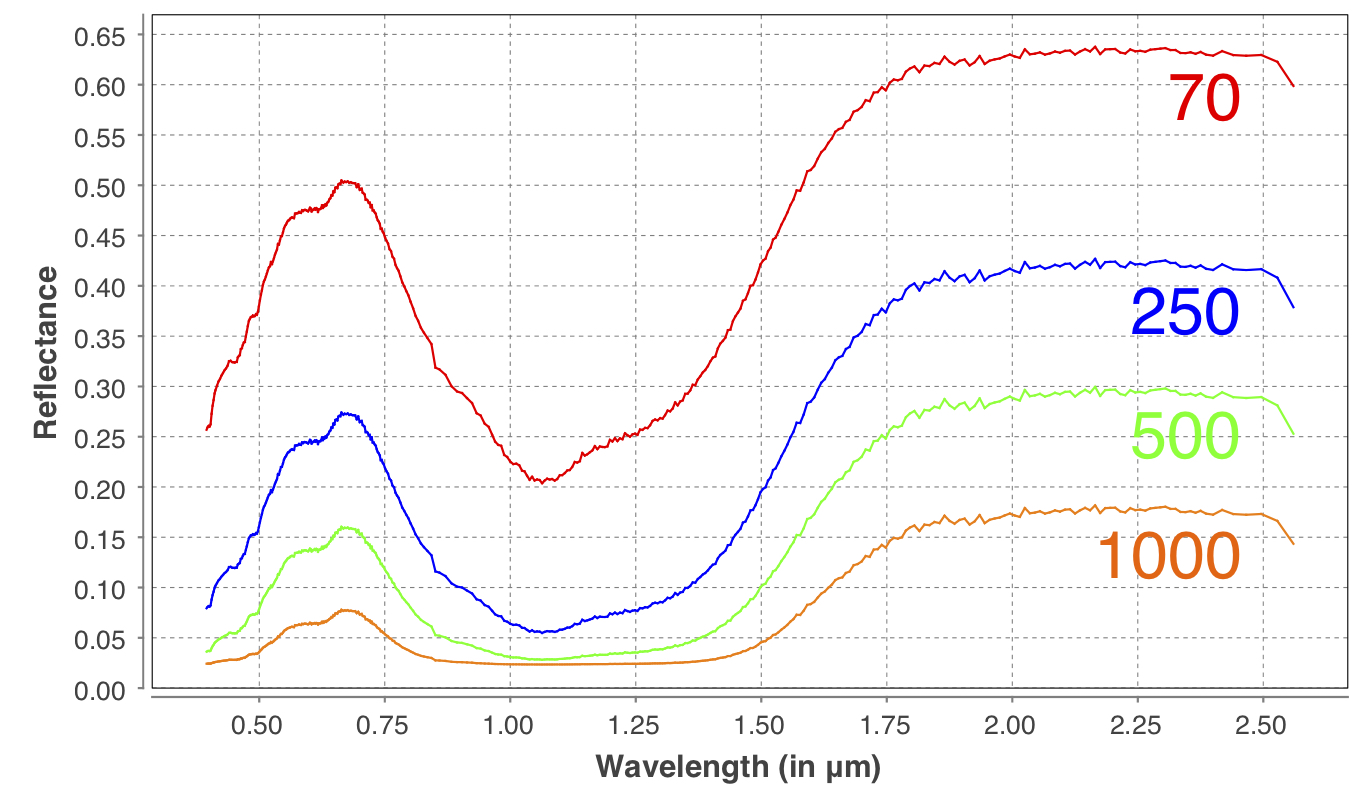


**Figure S.2**. Example Shkuratov model spectra of KI3189 used to prepare Figure 16 in the text, with grain sizes varying from 70 microns to 1mm.

**6. Carbonate Comparison map**

In order to provide another check that the 2.5 μm band that we have used to infer the presence of carbonates in our results and discussion in the main paper, we have prepared a composite image that is designed to light up only when the 2.3 and 2.5 μm bands are present. In Figure S.3, we have put the CAR image from Figure 6d of the text, and alongside it, a RGB composite map using a combination of summary products from Viviano-Beck et al. (2014). This particular image has been assigned these summary products to the RGB bands:

R: MIN2295_2480

G: MIN2345_2537

B: CINDEX2

In these colors, the carbonates show up in red/magenta/yellow/green colors. Comparing this to the CAR (Figure 6d) image, we can see that the carbonate locations (yellow-white) match up with the locations of carbonate detections in Figure S.5. This provides reassurance that the 2.5 μm band we have mapped is due to carbonates and not due to phyllosilicates or sulfates.


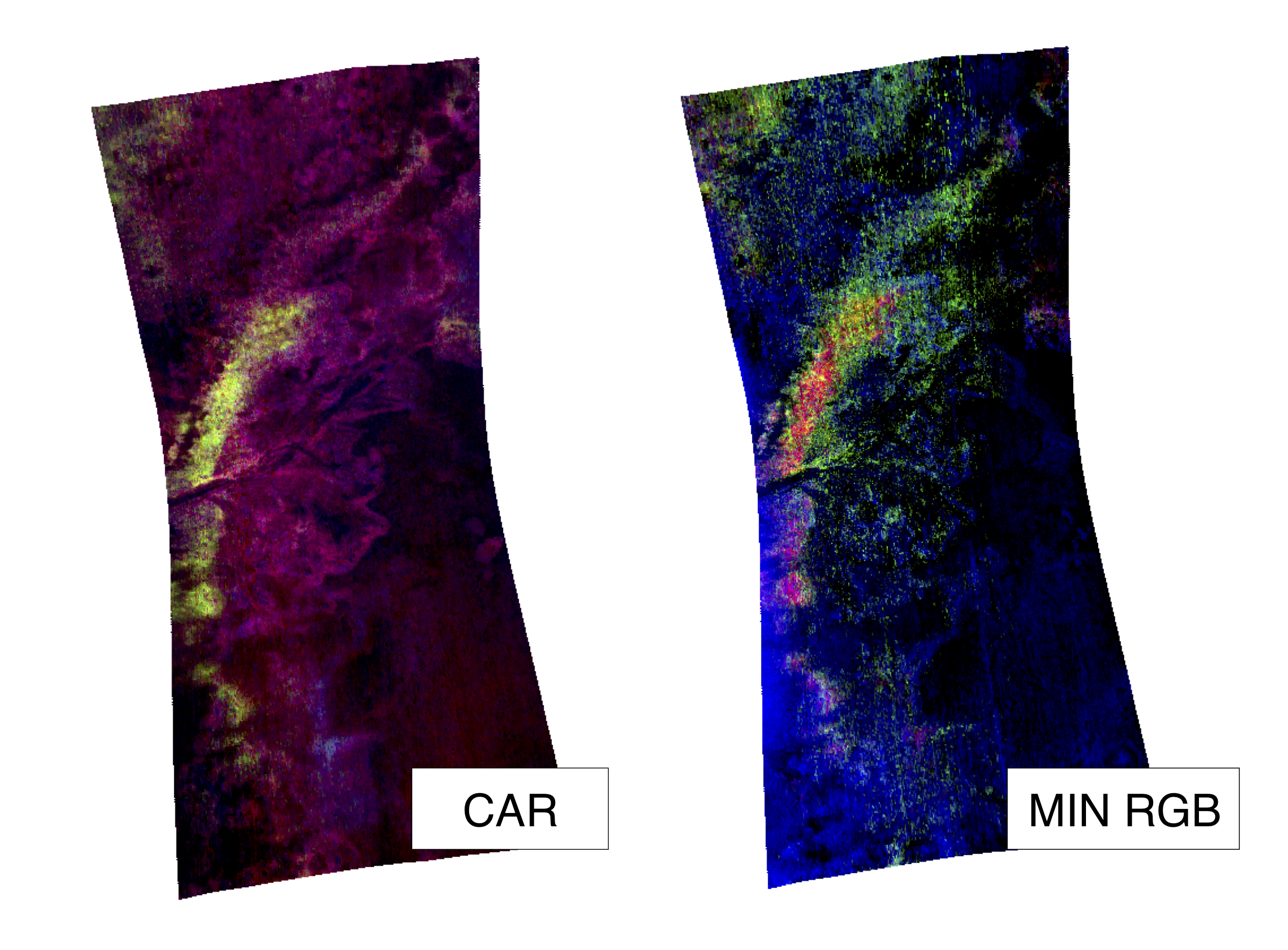


**Figure S.3**. (left) Figure 6d from the manuscript, showing the CAR false color image which highlights carbonates in yellow/white (right) False color image of CRISM FRT40FF showing an RGB composite designed to check the presence of 2.3 and 2.5 μm bands.

**References**

Bishop, J.L., J.J. Wray, B.L. Ehlmann, A.J. Brown, and M. Parente. “Refining Martian Carbonate Chemistries Determined through CRISM Analyses of Several Carbonate-Bearing Outcrops,” Abstract #2555. Houston, TX: LPI, 2013.

Brown, Adrian J. “Spectral Curve Fitting for Automatic Hyperspectral Data Analysis.” *IEEE Transactions on Geoscience and Remote Sensing* 44, no. 6 (2006): 1601–8. https://doi.org/10.1109/TGRS.2006.870435.

Brown, Adrian J., Simon J. Hook, Alice M. Baldridge, James K. Crowley, Nathan T. Bridges, Bradley J. Thomson, Giles M. Marion, Carlos R. de Souza Filho, and Janice L. Bishop. “Hydrothermal Formation of Clay-Carbonate Alteration Assemblages in the Nili Fossae Region of Mars.” *Earth and Planetary Science Letters* 297 (2010): 174–82. <https://doi.org/10.1016/j.epsl.2010.06.018>.

Clark, R.N., T. V. V. King, and N. Gorelick. “Automatic Continuum Analysis of Reflectance Spectra,” 138–42. Pasadena, CA: JPL Publication 87-30, 1987.

Corrigan, Catherine M., Timothy J. McCoy, Jessica M. Sunshine, S.J. Bus, and A. Gale. “Does Spectroscopy Provide Evidence for Widespread Partial Melting of Asteroids?: I. Pyroxene Compositions,” abstract 1463. Houston, TX: LPI, 2007.

Freeman, William, J. Bishop, F. Marchis, J. Emery, A. E. Reiss, T. Hiroi, D. Barrado y. Navascués, M. H. Shaddad, and P. Jenniskens. “Investigation of the Origin of 2008TC3 Through Spectral Analysis of F-Type Asteroids and Lab Spectra of Almahata Sitta and Mineral Mixtures.” *Bulletin of the American Astronomical Society* 42 (October 1, 2010): 13.31. <http://adsabs.harvard.edu/abs/2010DPS....42.1331F>.

King, T. V. V., and W. I. Ridley. “Relation of the Spectroscopic Reflectance of Olivine to Mineral Chemistry and Some Remote Sensing Implications.” *Journal of Geophysical Research* 92 (1987): 11457–69.

Murchie, S., R. Arvidson, P. Bedini, K. Beisser, J.-P. Bibring, J. Bishop, J. Boldt, et al. “Compact Reconnaissance Imaging Spectrometer for Mars (CRISM) on Mars Reconnaissance Orbiter (MRO).” *Journal of Geophysical Research* 112, no. E5 (2007): E05S03, doi:10.1029/2006JE002682. 10.1029/2006JE002682.

Mustard, J.F., and C.M Pieters. “Photometric Phase Functions of Common Geologic Minerals and Applications to Quantitative Analysis of Mineral Mixture Reflectance Spectra.” *Journal of Geophysical Research* 94 (1989): 13619–34.

Nelder, J.A., and R. Mead. “A Simplex Method for Function Minimization.” *Computer Journal* 7 (1965): 308–13

Rossman, G.R. “Vibrational Spectroscopy of Hydrous Components.” *Hawthorne, F.C., Ed., Spectroscopic Methods in Mineralogy and Geology: Mineralogical Society of America Reviews in Mineralogy* 18 (1988): 193–206.

Savitzky, A., and M.J.E. Golay. “Smoothing and Differentiation of Data by Simplified Least Squares Procedures.” *Analytical Chemistry* 36 (1964): 1627–39.

Seelos, F. P., M. F. Morgan, H. W. Taylor, S. L. Murchie, D. C. Humm, K. D. Seelos, O. S. Barnouin, C. E. Viviano, and CRISM Team (2012), CRISM Map Projected Targeted Reduced Data Records (MTRDRs) – High Level Analysis and Visualization Data Products, in Planetary Data: A Workshop for Users and Software Developers, Flagstaff, AZ

Sunshine, J. M., and C. M. Pieters. “Determining the Composition of Olivine from Reflectance Spectroscopy.” *Journal of Geophysical Research-Planets* 103, no. E6 (June 25, 1998): 13675–88. :[//000076969700005](file://000076969700005/).

Viviano-Beck, Christina E., Frank P. Seelos, Scott L. Murchie, Eliezer G. Kahn, Kimberley D. Seelos, Howard W. Taylor, Kelly Taylor, et al. “Revised CRISM Spectral Parameters and Summary Products Based on the Currently Detected Mineral Diversity on Mars.” *Journal of Geophysical Research: Planets*, June 1, 2014, 2014JE004627. https://doi.org/10.1002/2014JE004627.
